# Optimal supply chains and power sector benefits of green hydrogen

**DOI:** 10.1038/s41598-021-92511-6

**Published:** 2021-07-09

**Authors:** Fabian Stöckl, Wolf-Peter Schill, Alexander Zerrahn

**Affiliations:** 1 German Institute for Economic Research (DIW Berlin), Berlin, Germany; 2 Technische Universität Berlin, Berlin , Germany; 3 Climate & Energy College, Energy Transition Hub, University of Melbourne, Melbourne, Australia

**Keywords:** Energy infrastructure, Energy storage, Renewable energy

## Abstract

Green hydrogen can help to decarbonize parts of the transportation sector, but its power sector interactions are not well understood so far. It may contribute to integrating variable renewable energy sources if production is sufficiently flexible in time. Using an open-source co-optimization model of the power sector and four options for supplying hydrogen at German filling stations, we find a trade-off between energy efficiency and temporal flexibility. For lower shares of renewables and hydrogen, more energy-efficient and less flexible small-scale on-site electrolysis is optimal. For higher shares of renewables and/or hydrogen, more flexible but less energy-efficient large-scale hydrogen supply chains gain importance, as they allow to temporally disentangle hydrogen production from demand via storage. Liquid hydrogen emerges as particularly beneficial, followed by liquid organic hydrogen carriers and gaseous hydrogen. Large-scale hydrogen supply chains can deliver substantial power sector benefits, mainly through reduced renewable curtailment. Energy modelers and system planners should consider the distinct flexibility characteristics of hydrogen supply chains in more detail when assessing the role of green hydrogen in future energy transition scenarios. We also propose two alternative cost and emission metrics which could be useful in future analyses.

## Introduction

The increasing use of renewable energy sources in all end-use sectors is a main strategy to reduce greenhouse gas emissions^1^. This not only applies to the power sector, but also to other sectors such as transportation. There, energy demand may be satisfied either directly by renewable electricity or indirectly by hydrogen and derived synthetic fuels produced with renewable electricity^2–6^. The potential role of hydrogen-based electrification for deep decarbonization is widely acknowledged^7–10^.

Yet, a central aspect is less understood so far: how hydrogen-based electrification interacts with the power sector. Hydrogen supply chains use different types of storage, which allow to temporally disentangle electricity demand for hydrogen production from the time profile of final hydrogen demand. Similar to other flexibility options in the power sector, such as load shifting or electricity storage, this increases the *temporal flexibility* of the power sector. Such flexibility can help make better use of variable renewable energy from wind and solar PV^11,12^. This, in turn, impacts the optimal electricity generation and storage capacities in the power sector, their hourly use, carbon emissions, and costs. Yet more flexible hydrogen supply chains may be less energy-efficient as they incur more conversion steps^13,14^. Thus, the overall power system impacts of different hydrogen supply chains, considering both their flexibility and energy efficiency characteristics, are a priori unclear.

We address this research gap on the power sector interactions of green hydrogen by investigating different supply chains of hydrogen for road-based passenger mobility for future scenarios with high shares of variable renewable electricity. Specifically, we determine least-cost options for the supply of electrolysis-based hydrogen at filling stations, while explicitly considering how they interact with the power sector. To this end, we use an open-source cost-minimization model with a technology-rich well-to-tank perspective that co-optimizes the power sector and four relevant hydrogen supply chains derived from the literature: small-scale on-site electrolysis at the filling station as well as three large-scale hydrogen production and distribution options.

As outlined in more detail in “Literature review”, many previous power sector analyses that include hydrogen for mobility lack detail with respect to the representation of hydrogen production and distribution options^15–19^. In contrast, studies that include more techno-economic details of supply chains for hydrogen mobility often rely on exogenous electricity price inputs, include only rudimentary power sectors, tie hydrogen production to the availability of surplus electricity generation, and/or are restricted to a single supply chain^13,14,20–25^. Yet, none of these studies examines the interactions between hydrogen supply chains and power sectors with high shares of renewable energy sources in detail.

In this paper, we develop and apply an integrated hydrogen and power sector model to fill this gap in the literature. It minimizes overall system costs by endogenously optimizing electricity generation and storage capacities, their hourly dispatch, as well as capacity and hourly use of hydrogen supply chains. We parameterize our model to a 2030 setting for Germany. The insights derived for this case study should also be of interest to a range of other countries (see “Discussion”). Germany’s power sector is the largest in Europe. Traditionally, it has been dominated by thermal power plants, and is now increasingly shifting towards variable renewable energy sources, as dispatchable renewable sources such as hydro or geothermal energy are limited. Since the first version of the Renewable Energy Sources Act (EEG) entered into force in 2000, the German government repeatedly committed itself to an ambitious expansion of renewable energy sources^26^. This has put Germany among the global front-runner countries in terms of variable renewable energy use^27^. Recently, the German government also substantially increased its ambition to use green hydrogen and aims to become a major supplier of green hydrogen technologies^28^.

## Literature review

The existing literature covering the use of hydrogen in the mobility sector can be roughly divided into two groups based on their focus. The first group of analyses focuses on decarbonizing the power sector or even the whole economy, but models the use of hydrogen only in a very stylized way. The second group focuses on a detailed techno-economic representation of different hydrogen production and distribution schemes, but not on their interaction with the power sector.

The first group, which focuses on decarbonization, comprises, for instance, an analysis of Breyer et al.^16^. The authors model a generic $\hbox {H}_{{2}}$ demand for mobility (road, marine, aviation, rail) within a worldwide 100 % renewable energy setting for 2050. They find a share of hydrogen accounting for about 25 % of total energy demand in the transportation sector. Yet, as the study covers energy systems on a global scale, it does not provide great temporal and technological detail of hydrogen production and distribution. The same is true for Bogdanov et al.^15^. There, the authors apply a similar model to the case of Kazakhstan, an extreme example with unfavorable climatic conditions and an energy-intensive industry that make decarbonization with renewable energy sources more challenging than elsewhere. Similarly, Gils and Simon^18^ develop a 100 % renewable energy scenario for the Canary Islands, with hydrogen powering between 37 % and 75 % of road transportation. The authors find that the use of hydrogen may provide additional flexibility and facilitate the integration of high shares of renewables. However, their model only features a stylized hydrogen sector with a generic storage option. Brown et al.^17^ compare the effect of increased coupling of the power, heat, and transportation sectors vis-à-vis an extension of electricity transmission networks for high shares of renewables in Europe. Modeling different fleets of fuel-cell electric vehicles (FCEV), the authors find that the availability of large-scale storage can make hydrogen an important flexibility option in the power sector. Yet, due to its high temporal and spatial resolution, only a simplified hydrogen supply system is modeled. Finally, Oldenbroek et al.^19^ analyze 100 % renewable energy scenarios with fuel-cell electric vehicles that also feature vehicle-to-grid support for several European countries. They find that the backup power provided by a share of 50 % of cars being grid-connected FCEVs is sufficient to balance the power system. However, the hydrogen sector modeling does not account for different production and distribution chains. While all these studies have their merits, none provides sufficient techno-economic hydrogen sector details for an in-depth analysis of how different hydrogen production and distribution options interact with the power sector.

In contrast to the studies mentioned above, the second group of literature focuses on highly detailed representations of the hydrogen sector. For instance, Welder et al.^25^ and Samsatli et al.^24^ analyze hydrogen-to-mobility for Germany and Great Britain, respectively, but do not connect hydrogen production to the power system. Instead, electricity demand for electrolysis is covered by a wind power capacity built and used exclusively for that purpose. Moreover, the transmission of hydrogen is restricted to pipelines. The authors find $$\hbox {H}_{{2}}$$ to be cost-competitive with fossil fuels in the transportation sector^25^, and that all of Great Britain’s domestic transport can be supplied by onshore wind-powered hydrogen production^24^. In a similar study, Robinius et al.^22^ restrict electrolysis to be powered only by renewable surplus energy, which is derived for a predetermined capacity in a German 2050 scenario. Again, hydrogen is assumed to be transported to filling stations via pipelines. The authors find that renewable surplus electricity would be sufficient to serve Germany’s hydrogen demand for mobility. However, the research design neglects the effects hydrogen production may have on the power sector. Studying hydrogen-for-mobility pathways in Germany, Emonts et al.^20^ consider various options for hydrogen distribution and storage. Again, the electricity demand for water-electrolysis is constrained to be served by renewable surplus generation within a predetermined future energy system. The authors identify pipeline transmission as cost-optimal for large demands of hydrogen, while transportation via trucks is more favorable for lower demands. Taking the view of a wind turbine operator, Glenk and Reichelstein^21^ compare the grid feed-in of wind energy with its alternative use for hydrogen production. They find that hydrogen production is currently more profitable if it can be sold at prices of  3.23 €/kg (Germany) and  3.53 $/kg (Texas). The analysis abstracts from power sector modeling and instead draws on past electricity prices. Yang and Ogden^14^ and Reuß et al.^13^ compare several highly detailed hydrogen production and distribution chains. They find that the cost-optimal supply chain mainly depends on the average transportation distance and on overall hydrogen demand. Yet again, their analyses rely on exogenous electricity price assumptions. In contrast, Runge et al.^23^ use an electricity market model to derive hourly electricity prices for different market designs to analyze the costs of hydrogen supply based on liquid organic hydrogen carriers at German filling stations. Still, their model setup covers only the effect of electricity prices on the hydrogen sector, but not the feedback in the other direction. While all analyses of the second group represent the hydrogen sector with high techno-economic detail, they do not allow for investigating its interaction with the power sector.

There are only a few studies that explicitly account for such interactions between the two sectors. These are located between the two groups sketched above and conceptually closest to our work. Despite uncovering important new insights, in general, they are characterized by an incomplete co-optimization of sectors or a lack of detail in the representation of hydrogen production and distribution. For instance, Michalski et al.^29^ investigate the impact of different availabilities of hydrogen mobility infrastructure on German power plant dispatch. In contrast to our study, hydrogen infrastructure, as well as large parts of electricity generation capacities, are exogenously set and not the result of a co-optimization of the two sectors. Moreover, while the authors allow for both centrally produced hydrogen close to large underground storage (salt caverns) and on-site production at filling stations, the share of either type is not endogenous, but fixed. Results indicate that large-scale cavern storage allows for flexible deployment of electrolysis capacities, thus reducing the curtailment of wind and solar power. In another study, Rose and Neumann^30^ optimize the spatial distribution and storage size of hydrogen filling stations for heavy-duty trucks in Germany. They show that co-optimization with respect to total system cost, i.e., including the power sector and local grid restrictions, can reduce costs of hydrogen provision by up to 10 % compared to a non-optimized spatial distribution pattern. This cost reduction is mainly driven by lower electricity costs, as hydrogen production is better aligned with the sector’s flexibility needs. However, their analysis only considers on-site electrolysis at filling stations and thus cannot account for potential trade-offs between different hydrogen production and distribution options. Finally, Zhang et al.^31^ investigate the effect of flexible hydrogen production for mobility on the Western U.S. power system. They find a trade-off between the benefits of flexible hydrogen production for the power system operation and the expenditures for respective infrastructure costs, i.e., additional electrolyzer and storage capacity. In their model, a minimum of total costs is reached for slightly oversized electrolyzers in the range of 11 to 25 % compared to a scenario with flat production and a capacity factor of 100 %. However, their analysis is based on a very stylized hydrogen sector and lacks a representation of the specific characteristics of different hydrogen distribution pathways. Moreover, the authors focus on the dispatch of existing generation capacities and neglect the effects of hydrogen on optimal capacity expansion.

Overall, the review of the existing literature reveals a lack of detail in the representation of either the hydrogen or the power sector, and/or a missing co-optimization of both sectors. This impedes a thorough analysis of potential benefits and challenges related to the interaction of variable renewables and different hydrogen production and distribution options. Our analysis adds to the literature by providing, to the best of our knowledge, the first full co-optimization of different hydrogen supply chains and the power sector. Thereby, we consider a range of scenarios in which we systematically vary assumptions on future hydrogen demand and the share of renewable energy sources. Specifically, we investigate the trade-off between energy efficiency and temporal flexibility for different hydrogen supply chains, and how it interacts with optimal capacity and dispatch outcomes in the power sector.

## Model and scenarios

### The open-source power sector model DIETER

We use the established open-source power sector model DIETER. Different versions of this model have been previously used for analyzing aspects of renewable energy integration with a focus on utility-scale energy storage^32–34^, decentralized storage related to prosumers^35,36^, and power-to-heat options^37^. The model includes several features essential for meaningful analyses of integrating variable renewable energy sources, in particular a sufficient temporal resolution^38,39^ and a detailed modeling of energy storage^40^. For transparency and reproducibility^41^, the source code, input data, and a complete documentation of the model version used here are available under a permissive open-source license in a public repository^42^ (see also www.diw.de/dieter).

The model minimizes the total system costs of providing electricity and hydrogen. The objective function comprises annualized investment costs and hourly variable costs of electricity generation and storage technologies, electrolysis, as well as storage, conversion, and transportation of hydrogen. The main model inputs are availability and cost parameters for all technologies as well as hourly time series of electricity demand, hydrogen demand, and renewable capacity factors. The main decision variables are capacities in the power and hydrogen sectors as well as their hourly use. The optimization is subject to constraints, including market balances for electricity and hydrogen that equate supply and demand in each hour, capacity limits for generation and investment, and a minimum share of renewable energy in electricity supply. The model determines a long-run first-best equilibrium benchmark for a frictionless market. Assuming perfect foresight, DIETER is solved for all consecutive hours of an entire year, thereby capturing the variability of renewable energy sources. Model outputs comprise system costs, optimal capacities and their hourly use, and derived metrics such as emission intensities.

The electricity demands of various processes along the hydrogen supply chains enter the model’s energy balance. This includes electricity used for hydrogen production, processing, and distribution facilities. Depending on the conversion steps along the supply chain, the four options differ in how much electricity is required overall, and at which stage of the process (for an illustration, see Sect. SI.4). All costs for hydrogen-related investments enter the model’s objective function.

As we aim to derive general insights on temporal flexibility, we abstract from an explicit representation of idiosyncratic spatial aspects and electricity network constraints. Moreover, to keep the analysis tractable, the DIETER version used here has no explicit representation of electricity transmission, focuses on Germany only, and abstracts from balancing within the European interconnection. We also do not use some features of the original model, such as demand-side flexibility beyond the hydrogen sector.

### Four hydrogen supply chains

**Figure 1:**
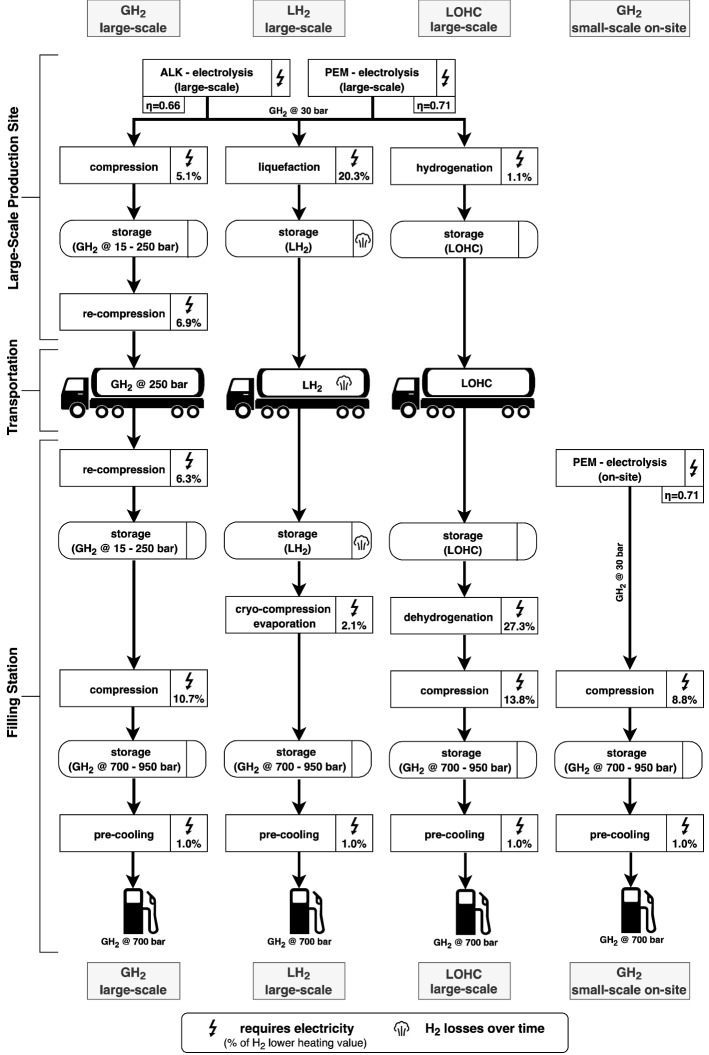
Large-scale and small-scale on-site supply chains with specific production, processing, transportation, and storage requirements.

The hydrogen sector is modeled with a well-to-tank perspective. It includes four options to provide filling stations with hydrogen: small-scale on-site electrolysis directly at the filling station, and three more centralized large-scale options, where $\hbox {H}_{{2}}$ is delivered by trailers (Fig. 1). The three centralized supply chains are adapted from previous analyses of hydrogen production and distribution^13,14^ and are characterized by the availability of large-scale hydrogen generation and storage. They mainly differ with respect to the form in which hydrogen is stored: gaseous hydrogen ($\hbox {GH}_2$), liquid hydrogen ($\hbox {LH}_2$), or bound to a liquid organic hydrogen carrier (LOHC, see^43^). In contrast, on-site hydrogen production, which leans on^29^, comes with only limited amounts of hydrogen storage in high-pressure gas tanks, which is motivated by space and security reasons. For our analysis, only one supply chain can be selected per filling station.

Small-scale on-site hydrogen production is restricted to proton exchange membrane (PEM) water electrolysis, which is superior to alkaline (ALK) electrolysis in several dimensions relevant for small-scale on-site production, including higher load flexibility^44^, a lower footprint^44^, and easier handling^45^. Locally produced hydrogen is immediately compressed and stored at $700-950$ bar in high-pressure vessels at the filling station. The same high-pressure storage and dispensing installations are also present in the large-scale supply chains.

For large-scale hydrogen production, we consider both ALK and PEM electrolysis. As the large-scale options allow for bulk hydrogen storage, they provide greater temporal flexibility compared to the small-scale on-site option, which only comes with a short-term buffer storage at the filling station. Hydrogen from electrolysis is either compressed and stored at the production site at up to 250 bar ($\hbox {GH}_2$), liquefied and stored in insulated tanks ($\hbox {LH}_2$), or bound to a liquid organic hydrogen carrier (LOHC) in an exothermic hydrogenation reaction and stored in simple tanks. As LOHC, we assume dibenzyltoluene; see^46^ for an exposition. $\hbox {GH}_2$ and LOHC can be stored without losses; $\hbox {LH}_2$ suffers from a boil-off of $\sim$ 0.2 % per day ($\sim$ 52 % per year), which lowers its potential for long-term $\hbox {H}_2$ storage. For $\hbox {GH}_2$, hydrogen may also be directly prepared for transportation after production, bypassing production-site storage. Investments in storage capacity at large-scale production sites are unrestricted. Due to minimum filling level requirements, usable storage capacities can be lower than nominal capacities.

For transportation, hydrogen is taken from the respective storage at the large-scale production site, re-compressed (if necessary), and transported (time-consuming) in special trailers to the filling stations.

At filling stations, $\hbox {GH}_2$ from large-scale electrolysis is either re-compressed and stored at up to 250 bar or directly compressed to 950 bar for the high-pressure buffer storage (bypass option). $\hbox {LH}_2$ and LOHC are first stored in unconverted form, where boil-off for $\hbox {LH}_{{2}}$ is slightly higher at the filling station than at the large-scale production site ($\sim$ 0.4 % per day or $\sim$ 77 % per year). Spatial limitations and security aspects restrict these storage capacities to two trailer-loads for all three large-scale supply chains. $\hbox {LH}_2$ is then cryo-compressed and evaporated, and LOHC dehydrogenated and compressed to be stored in gaseous form at up to 950 bar in high-pressure vessels used as a buffer for dispensing. High-pressure storage is limited to 300 kg (one 20 ft container with tubes^47^).

### Renewable energy share and hydrogen demand scenarios

Twelve scenarios vary the share of renewable energy sources in electricity generation between 65-80 % in five percentage point increments, and the demand for hydrogen between 0, 5, 10, and 25 % of private and public road-based passenger vehicle energy demand. A renewable share of 65 % exactly matches the current German government’s target for 2030. Larger shares reflect higher ambition levels, which Germany aims for beyond 2030, and are required for achieving more progressive climate targets. We abstract from modeling renewable shares beyond 80 % here, as these appear to be more plausible in longer-term settings in which other sector coupling technologies and the reconversion of hydrogen to electricity would also become more relevant. Note that increasing the share of renewable energy beyond 65 % requires additional deployment of variable solar PV or wind power capacity, as the potentials of dispatchable hydro and bioenergy sources are already fully realized.

Annual hydrogen demands are 9.1, 18.1, and 45.3 $\hbox {TWh}_{H_{2}}$ at the filling stations, representing different potential future market penetrations of hydrogen-electric mobility. This relates to 5 %, 10 %, or 25 % of road-based passenger traffic in Germany (compare Sect. SI.4.2). These hydrogen demands substantially exceed those of the fleet of fuel cell electric vehicles that can be reasonably expected in 2030. Yet, the demand levels used here allow for interesting insights in settings where hydrogen demand is non-negligible from a power sector perspective. For clarity, we abstract from the provision of hydrogen for other purposes than mobility.

For each renewable energy share and hydrogen demand scenario, we combine the small-scale on-site hydrogen supply option with either of the three large-scale options. This results in three distinct combinations of options per scenario. Due to path dependencies and technology specialization, we do not expect parallel infrastructures for large-scale technologies to emerge in a plausible future setting.

## Results

### Optimal hydrogen supply chains depend on renewable penetration and hydrogen demand

**Figure 2:**
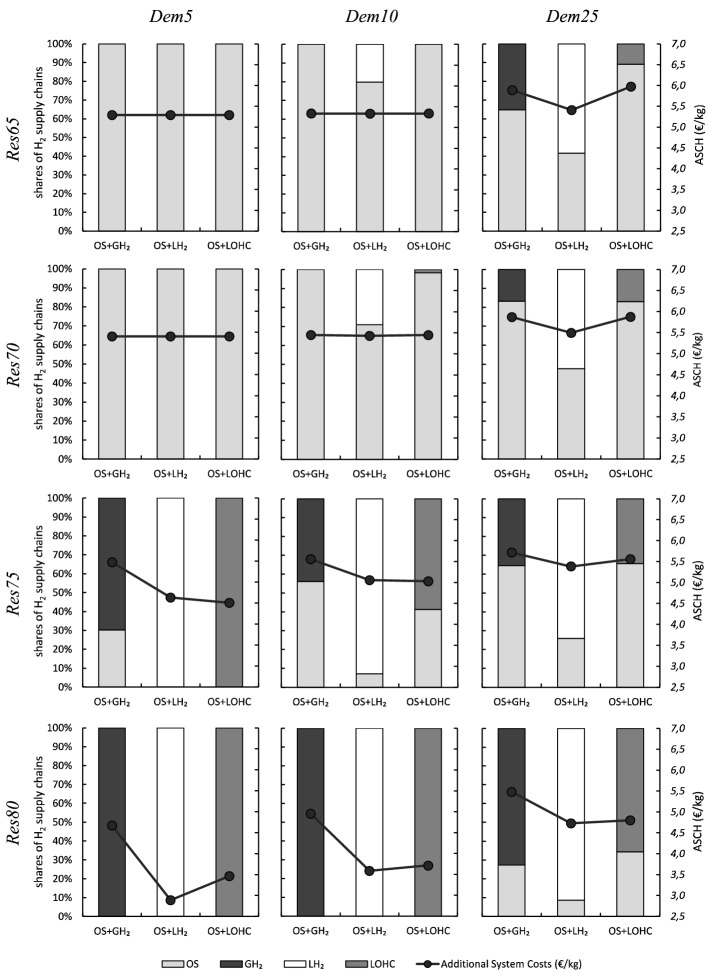
Optimal combinations of small-scale on-site (OS) and large-scale hydrogen supply chains and Additional System Costs of Hydrogen (ASCH) for the 12 scenarios. Starting from the top left panel, the share of renewable energy sources increases to the bottom, and the demand for hydrogen increases to the right.

Figure 2 shows the cost-minimal combinations of small-scale on-site (OS) and large-scale hydrogen supply chains for the 12 scenarios with hydrogen demand. We denote the resulting renewables-demand scenarios as *Res*65-*Dem*5, *Res*65-*Dem*10, and so on. The Figure also shows the Additional System Costs of Hydrogen (ASCH, see also Sect. SI.1), defined as difference in total system costs between a scenario that includes hydrogen and the respective baseline without hydrogen demand, related to total hydrogen supply.

For combinations of relatively low shares of renewable energy sources (65-70 %) and hydrogen demand (5-10 % of road-based passenger traffic), small-scale electrolysis is the least-cost option. That is, the energy efficiency benefits of on-site electrolysis prevail over the flexibility benefits of large-scale options. Large-scale supply chains are increasingly part of the optimal solution for higher shares of renewables or greater hydrogen demand. In these scenarios, the flexibility they offer becomes more valuable. Among the three large-scale options, liquid hydrogen tends to have the highest shares in the optimal solution.

Comparing the Additional System Costs of Hydrogen, the solutions that include compressed gaseous hydrogen are always dominated by liquid hydrogen and often also by LOHC. This is because $\hbox {GH}_{{2}}$, while energy efficient, incurs comparably high storage and transportation costs (see Sect. SI.4). In contrast, solutions that include $\hbox {LH}_{{2}}$ lead to the lowest ASCH in most scenarios with high renewable shares (75-80 %) or high hydrogen demand (25 %). In general, solutions that include $\hbox {LH}_{{2}}$ or LOHC often lead to relatively similar cost outcomes. Yet, this is driven by different underlying mechanisms. $\hbox {LH}_{{2}}$ is overall more energy efficient; LOHC offers higher temporal flexibility due to cheap storage, yet requires substantial amounts of electricity for the dehydrogenation process at the filling station (see “Use patterns of hydrogen production and storage indicate differences in temporal flexibility” and Sect. SI.4).

Further, the Additional System Costs of Hydrogen generally increase with hydrogen demand and decrease with the share of renewable energy sources, mainly reflecting the availability of cheap renewable surplus energy (see “Power sector outcomes reflect drivers for optimal hydrogen supply chains”).

### Use patterns of hydrogen production and storage indicate differences in temporal flexibility

Differences in hydrogen storage capabilities as well as the level and timing of electricity demand (Sect. SI.4) lead to very different utilization patterns of the four hydrogen supply chains. We illustrate this for the optimal combination of temporally inflexible small-scale electrolysis and more flexible $\hbox {LH}_{{2}}$ in the *Res*80-*Dem*25 scenario.

**Figure 3:**
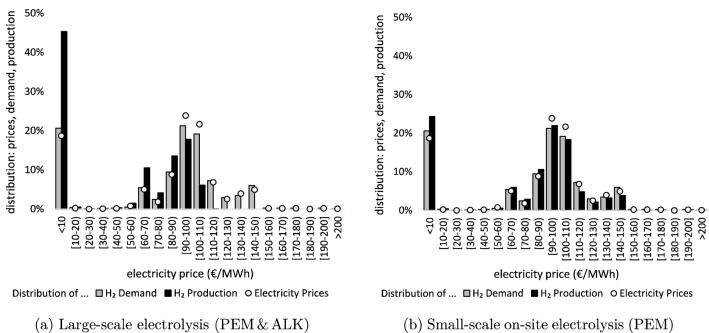
Distribution of hydrogen production, hydrogen demand, and electricity prices, exemplary for OS+$\hbox {LH}_{{2}}$ in scenario *Res*80-*Dem*25.

Figure 3a shows that $\hbox {LH}_{{2}}$ allows to temporally disentangle hydrogen production from demand. On average, production is high during hours when (renewable) electricity is abundant and, thus, cheap. These are not necessarily hours of high hydrogen demand. At the filling station, dispensing $\hbox {LH}_{{2}}$ on time requires only little electricity. Vice versa, large-scale hydrogen production is low during hours of high prices. In contrast, on-site electrolysis only includes a small high-pressure buffer storage and needs to produce almost on demand (Fig. 3b). Thus, through greater temporal flexibility, $\hbox {LH}_{{2}}$ allows to exploit phases of high renewable electricity supply and accordingly low electricity prices, which can overcompensate the overall higher electricity demand. Comparable production patterns also emerge for the other two large-scale supply chains $\hbox {GH}_2$ and LOHC.

**Figure 4:**
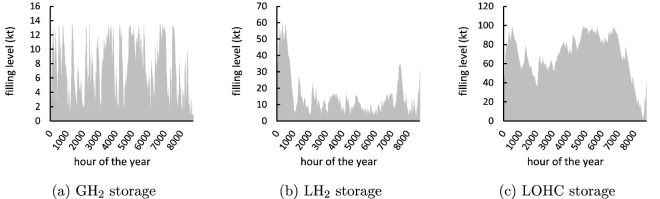
Temporal use pattern of production-site storage in scenario *Res*80-*Dem*25.

The capacities of production-site hydrogen storage and its hourly use vary substantially across the three large-scale options (Fig. 4). LOHC has the highest overall storage capacity and a strongly seasonal use pattern. In contrast, $\hbox {GH}_2$ has a much smaller storage capacity and a pronounced short-term storage pattern. $\hbox {LH}_2$ storage is in between. Capacity deployment of $\hbox {GH}_2$ storage is small because of its relatively high specific investment costs. This changes in a sensitivity with cheap cavern storage (see Sect. SI.2.3). For $\hbox {LH}_2$, storage investment costs are much lower, yet investment costs for liquefaction plants are high, impeding investments in larger $\hbox {LH}_2$ production capacities. $\hbox {LH}_2$ storage is also subject to a small, but relevant boil-off, which makes it less suitable for long-term storage. For LOHC, both investment costs for storage and hydrogenation plants are relatively low and investments, accordingly, high. As there is also no boil-off, LOHC storage is used for seasonal balancing.

### Power sector outcomes reflect drivers for optimal hydrogen supply chains

**Figure 5:**
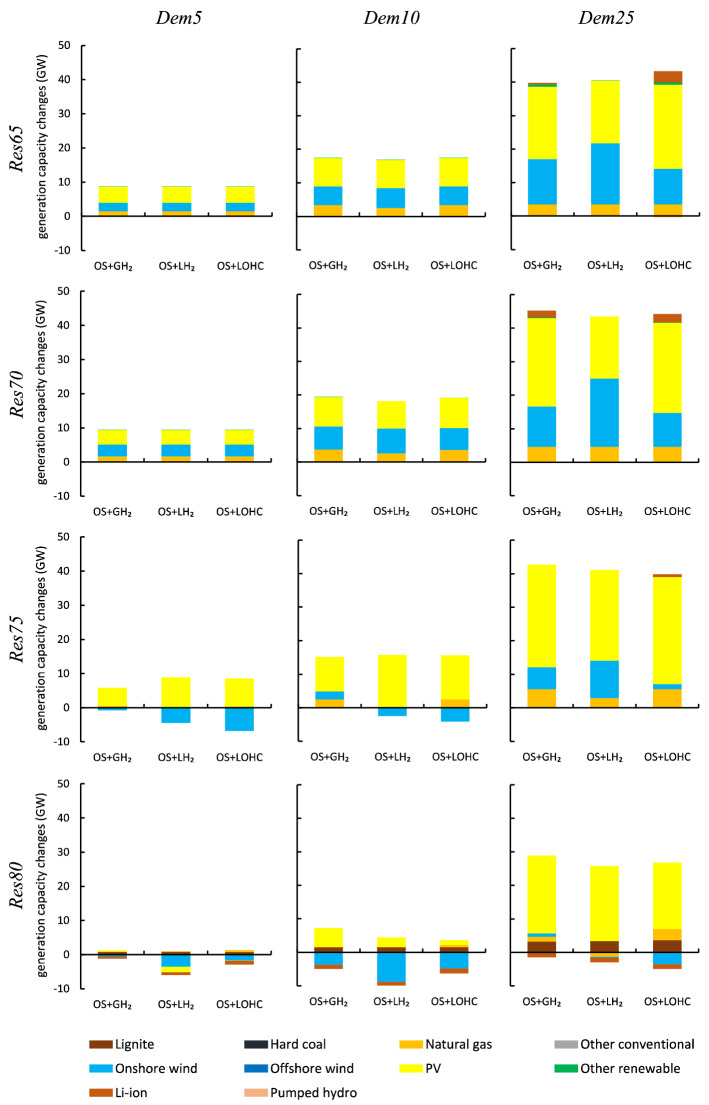
Electricity generation capacity changes compared to the respective baselines without hydrogen for optimal combinations of small-scale and large-scale hydrogen supply chains as shown in Fig. 2.

Figure 5 summarizes power sector capacity impacts for the scenarios. Each bar shows the difference of optimal generation capacities compared to the respective baseline without $\hbox {H}_2$ demand. Generally, overall generation capacity increases with growing hydrogen demand and decreases with growing renewable penetration. A higher renewable share leads to higher renewable surplus generation. Large-scale electrolyzers and storage make use of this surplus that would otherwise be curtailed. In fact, in scenarios *Res*80-*Dem*5 and *Res*80-*Dem*10, overall electricity generation capacity hardly increases or even decreases because the additional electricity demand for hydrogen production is covered by renewable electricity that would otherwise not be used.

Concerning specific technologies, the additional electricity demand for hydrogen supply yields larger optimal solar PV capacities. Additional investments in wind power are lower and the optimal wind power capacity even decreases in some *Res*75 or *Res*80 scenarios compared to the respective baseline. Additional wind power would lead to more sustained renewable surplus events, which would be harder to integrate. Offshore wind power is always deployed at the exogenous lower capacity bound of 17 GW. Further, we find a slight increase in the natural gas generation capacity in most scenarios because this is the most economical conventional generation technology to be operated with relatively low full-load hours. Compared to the respective baselines, the supply of hydrogen further tends to increase the optimal electricity storage capacity in the scenarios with lower renewable penetration because temporally inflexible on-site hydrogen production prevails here. In contrast, the optimal electricity storage capacity decreases in the *Res*80 scenarios. Here, large-scale hydrogen supply chains add a substantial amount of flexibility to the power sector.

**Figure 6:**
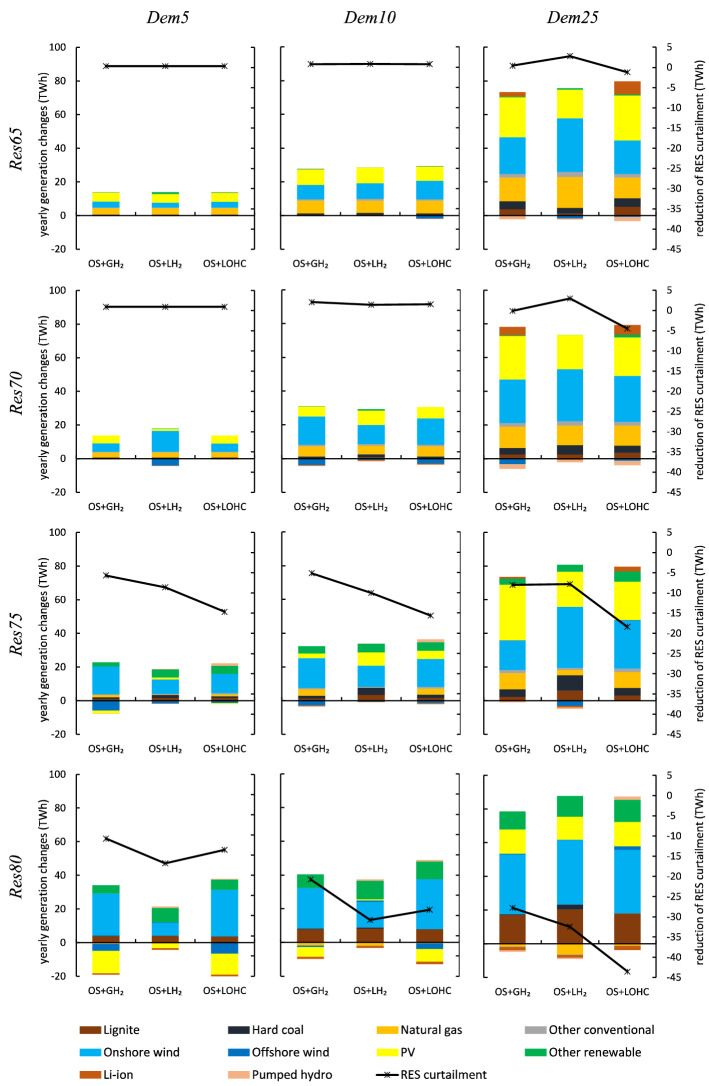
Yearly electricity generation changes compared to the respective baselines without hydrogen for optimal combinations of small-scale and large-scale hydrogen supply chains as shown in Fig. 2.

Figure 6 shows the impact of hydrogen supply chains on yearly energy generation. Across scenarios, wind power is a major source of the additional electricity required for hydrogen supply. Much of this wind power would be curtailed in a power sector without hydrogen. The central driver for this result is that large-scale hydrogen supply chains allow to make better use of variable renewable energy sources, facilitated through longer-term storage. In the *Res*75 and *Res*80 scenarios, electricity generation from wind turbines increases substantially although wind capacity barely increases or even decreases (compare Fig. 5). Renewable curtailment decreases most in scenario *Res*80-*Dem*25 with LOHC, where full-load hours of wind power increase by 19 %. LOHC has the largest capability to integrate renewable surpluses by means of storage and also requires the largest amount of electricity.

Power generation from conventional generators also increases and supplies the part of the additional electricity that is not covered by renewables according to the specified share. In the *Res*65-*Dem*25 and *Res*70-*Dem*25 scenarios, with largely inflexible, small-scale electrolysis, this is mainly natural gas-fired power generation. With increasing shares of renewable energy sources, there is a shift to hard coal and lignite. In *Res*80-*Dem*25, the share of lignite in non-renewable power generation is highest. Here, the temporal flexibility of large-scale hydrogen supply chains allows increasing the full-load hours of conventional generation with the highest fixed and lowest variable costs, i.e., lignite. Likewise, the use of electricity storage increases compared to the baseline in scenario *Res*65-*Dem*25, where inflexible small-scale on-site hydrogen supply prevails, but is substituted by large-scale hydrogen flexibility in scenario *Res*80-*Dem*25.

### $\hbox {CO}_{{2}}$ emission intensity of hydrogen may not decrease with higher renewable shares

**Figure 7:**
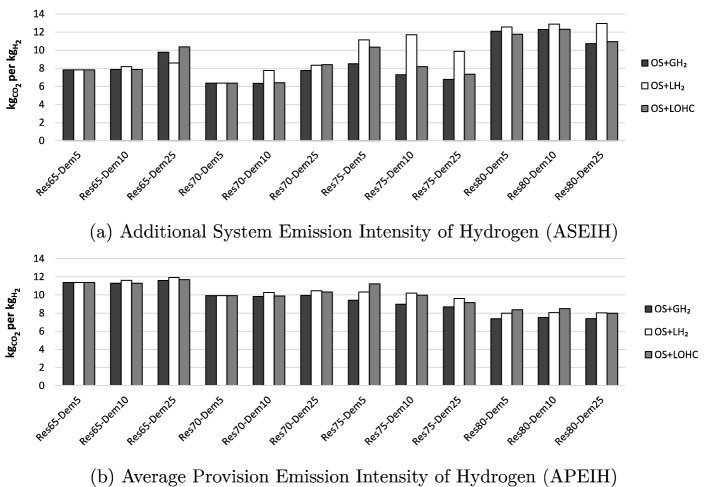
Emission metrics.

We calculate the $\hbox {CO}_2$ emission intensity of the hydrogen supplied in two complementary ways (see Sect. SI.1). The Additional System Emission Intensity of Hydrogen (ASEIH), shown in Fig. 7a, takes the full power sector effects of hydrogen provision into account. It is defined as the difference of overall $\hbox {CO}_2$ emissions between a scenario with hydrogen and the respective baseline without hydrogen, relative to the total hydrogen demand. The ASEIH mirrors the changes in yearly electricity generation induced by hydrogen supply and ranges between 6 and 13 kg $\hbox {CO}_2$ per kg $\hbox {H}_2$.

Among the *Res*65 scenarios, the emission intensity of hydrogen is higher for larger hydrogen demand (*Dem*25) because the greater role of flexible large-scale hydrogen infrastructure triggers an increase in coal-fired generation. For a renewable share of 70 %, the emission intensity is lower because overall power sector emissions decrease and the additional hydrogen demand largely integrates renewables without requiring additional fossil generation. In contrast, for high renewable shares of 75 % or 80 %, the ASEIH increases again because the flexibility related to the large-scale hydrogen supply chains allows integrating more coal-fired power generation. This is most pronounced for combinations of small-scale on-site electrolysis and $\hbox {LH}_2$, as the large-scale supply chain has a greater relevance in overall $\hbox {H}_2$ supply compared to OS+$\hbox {GH}_2$ or OS+LOHC. Under this metric, thus, the emission intensity of electrolysis-based hydrogen does not necessarily decrease with increasing renewable shares, absent further $\hbox {CO}_2$ regulation.

The second metric, Average Provision Emission Intensity of Hydrogen (APEIH), shown in Fig. 7b, does not capture the differences to an alternative power sector without hydrogen, but is based on $\hbox {CO}_2$ emissions prevailing in the hours of actual hydrogen production. The APEIH ranges between 7 and 12 kg $\hbox {CO}_2$ per kg $\hbox {H}_2$. The APEIH is highest for the *Res*65 scenarios and generally decreases with increasing renewable shares. It is lowest in supply chains with $\hbox {GH}_2$, slightly higher in with $\hbox {LH}_2$, and highest for LOHC. This largely reflects the differences in energy efficiency among these options.

For lower renewable shares, the APEIH tends to be higher than the ASEIH; for high renewable shares, the APEIH tends to be lower than the ASEIH. That is, a greater renewable penetration decreases the $\hbox {CO}_2$ emissions of the electricity mix used to produce hydrogen (APEIH), but additional emissions induced by $\hbox {H}_2$ do not necessarily decrease (ASEIH). This also indicates that analyses on the emission intensity of green hydrogen should generally be interpreted with care.

### Power sector benefits of hydrogen

**Figure 8:**
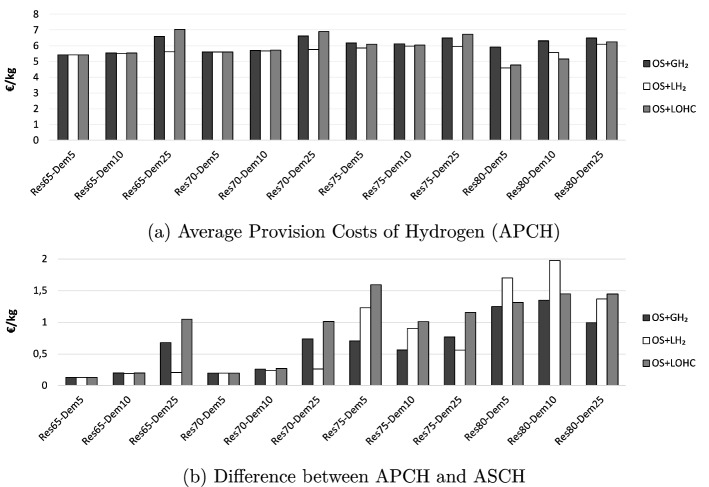
Average provision costs of hydrogen (APCH) and differences to additional system costs of hydrogen (ASCH).

We illustrate the power sector benefits of hydrogen supply in two different ways. First, the Average Provision Costs of Hydrogen (APCH) indicate hydrogen costs from a producer perspective. Across all scenarios, the APCH are between around 5 and 8 €/kg (Fig. 8a). These costs are below the uniform retail price of hydrogen in Germany of around 9.5 €/kg by 2020. In general, the APCH increase with hydrogen demand in all scenarios. With increasing shares of renewable energy, the APCH generally increase slightly, with the exception of scenarios *Res*80-*Dem*5 and *Res*80-*Dem*10. Here, supply chain combinations that include $\hbox {LH}_2$ or LOHC lead to lower costs because they can make better use of periods with very low electricity prices, which are frequent in this setting.

In contrast to APCH, the Additional System Costs of Hydrogen (ASCH) metric indicates the costs of hydrogen from a power system perspective. ASCH, which are also shown in Fig. 2, are smaller than APCH in all scenarios. This difference is substantially more pronounced for higher renewable shares (Fig. 8b). The ASCH also include the benefits of better renewable energy integration compared to a system without hydrogen. Yet, these benefits cannot be fully internalized by customers at filling stations, as the difference to the more production-oriented APCH metric indicates.

**Figure 9:**
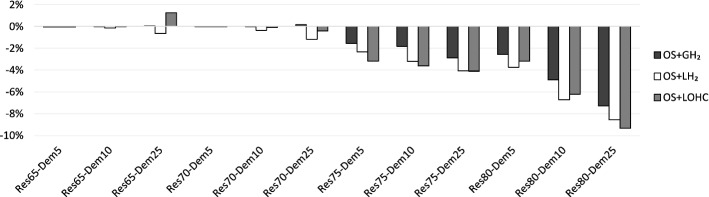
Effect of hydrogen on system costs of electricity (SCE).

Second, we illustrate the power sector benefits of different hydrogen supply chains with their impacts on the System Costs of Electricity (SCE, Sect. SI.1). Here, the total benefits of integrating the power and hydrogen sectors are attributed to the costs of generating electricity. For renewable shares of 65 % and 70 %, hydrogen hardly has an impact (Fig. 9). Yet, SCE decrease markedly for higher renewable shares, up to more than 9 % for a combination of small-scale on-site electrolysis and LOHC in the *Res*80-*Dem*25 scenario. The main driver for these benefits, again, is reduced renewable curtailment.

### Sensitivity analyses: impacts of central parameter assumptions on supply chains

Additional model runs show the impact of alternative assumptions for central input parameters (see Sect. SI.2). $\hbox {GH}_2$ and LOHC tend to improve relative to $\hbox {LH}_2$ if the transportation distance decreases, and vice versa, in particular if the share of large-scale production is high. If mass hydrogen storage could be placed at filling stations, this would greatly benefit the small-scale on-site supply chain. $\hbox {GH}_2$ becomes the dominant option for most scenarios if low-cost cavern storage can be developed. $\hbox {LH}_2$ would improve further if boil-off during storage could be avoided. In turn, LOHC would become dominant in most scenarios if free waste heat could be used for dehydrogenation, and if existing transportation and storage infrastructure could be used without additional costs.

## Qualitative effects of model limitations

Here, we briefly discuss some limitations of the study and how they may qualitatively impact results. Several research design choices we made for clarity and tractability lead to a power sector that is relatively flexibility-constrained. On the demand side, we abstract from a range of potential flexibility sources, such as power-to-heat options, battery-electric vehicles or the use of hydrogen for other purposes than mobility, e.g., high-temperature processes in industry. We also abstract from geographical balancing in the European interconnection. Accordingly, we may overestimate renewable surpluses and, in turn, the benefits of flexible hydrogen supply chains that make use of them. We also do not constrain investments in renewable electricity generation in Germany. A cap on renewable capacity deployment, reflecting public acceptance and planning issues, may further increase the relative importance of energy efficiency compared to flexibility.

We further do not consider potential transmission or distribution grid constraints for clarity and generalizability. These can increase the local value of flexible hydrogen supply, particularly in areas with very good renewable energy resources. For example, temporally flexible large-scale hydrogen supply chains may be particularly beneficial in Germany’s Northern region, where the best wind power resources are located.

Likewise, we abstract from hydrogen distribution via pipelines. These could resolve the efficiency-flexibility trade-off, but are likely to be economical only for transporting large amounts of hydrogen between major hubs.

## Discussion

Our co-optimization of the power and hydrogen sectors highlights that small-scale on-site electrolysis is most beneficial for lower shares of renewable energy sources and low hydrogen demand because energy efficiency matters more than temporal flexibility in such a setting. The power sector benefits of hydrogen are accordingly small. For higher shares of renewables or higher hydrogen demand, large-scale hydrogen infrastructure options gain importance. $\hbox {LH}_2$ provides the best combination of efficiency, flexibility, and investment cost over the majority of scenarios. In particular, temporally flexible large-scale supply chains make use of renewable surplus generation, which allows reducing optimal renewable capacity investment. Yet this flexibility not only facilitates renewable integration in the power sector, but can also increase the use of conventional generation with low marginal costs, with respective carbon emission effects (cp.^29^). The emission intensity of hydrogen thus not necessarily decreases with higher renewable shares, absent further $\hbox {CO}_2$ regulation.

Overall, the costs of supplying hydrogen at filling stations are relatively similar among optimal supply chain combinations in most of our modeled scenarios. Real-world investment choices would thus have to take additional factors into account that the model analysis cannot not capture. This includes aspects of operational safety and public acceptance, which may favor LOHC, or constraints to renewable energy deployment, which may favor the more energy-efficient options.

While cross-study comparisons are generally challenging due to differences in general model set-ups and parameterizations, our hydrogen cost results are largely in the range of previous analyses. Our Average Provision Costs of Hydrogen (APCH) of 5-8 €/kg are relatively similar to the values calculated by^29^ and^30^, and somewhat at the lower end of the values found by^13^ and^20^. Yet we go beyond the previous literature as our research design also allows for differentiating between APCH and Additional System Costs of Hydrogen (ASCH). The latter are generally lower, and increasingly so for higher shares of renewables, as they include the power sector benefits of better renewable integration. In addition to our numerical findings, our presentation and discussion of the APCH and ASCH metrics may be helpful for the energy modeling community. Similar cost calculations could also be carried out for other sector coupling options than green hydrogen. The same is true for the corresponding carbon emission metrics APEIH and ASEIH introduced here.

While our model analysis is parameterized for Germany, our main findings and conclusions should also apply to power sectors in other geographical settings that undergo a transformation from thermal generators toward variable renewable energy sources. Yet, in specific settings where large potentials of dispatchable renewable energy sources are available, such as hydro reservoirs and geothermal energy, the benefits of more flexible hydrogen supply chains are likely to be substantially lower than modeled here. Other countries may also differ from Germany with respect to important hydrogen-related factors such as typical transportation distances, the availability of low-cost hydrogen storage in caverns, the availability of low-cost waste or renewable heat sources for dehydrogenation, and the stock of existing transportation and storage infrastructure. The sensitivities presented in “Sensitivity analyses: impacts of central parameter assumptions on supply chains” qualitatively indicate how our model results depend on these factors.

We conclude that energy system analysts and planners should consider the flexibility and efficiency trade-off of green hydrogen in more detail when assessing its role in future energy transition scenarios. This requires a sufficiently detailed representation of hydrogen supply chains in respective energy modeling tools. To realize flexibility benefits in actual energy markets, policymakers should further redesign tariffs and taxes such that they do not overly distort wholesale price signal along all steps of the hydrogen supply chain (cp.^48^), while enabling a fair distribution of the benefits between hydrogen and electricity consumers.

Future research may aim to address some limitations of this study (cp. “Qualitative effects of model limitations”), or explore the efficiency-flexibility trade-off for different hydrogen carriers that allow long-range bulk transport of green hydrogen from remote areas with excellent wind or PV resources, such as Patagonia or Australia. Likewise, extending our analysis to also include the reconversion of hydrogen to electricity in scenarios with full renewable supply would be promising^5,49^.

## Supplementary Information

Supplementary Information.
