# Supplementary material for: Optimal supply chains and power sector benefits of green hydrogen
Source: Sci Rep. 2021 Jul 9;11:14191. doi: 10.1038/s41598-021-92511-6 (PMC8271012; doi:10.1038/s41598-021-92511-6)
Supplement: Supplementary file 1 — Supplementary Information. [file 41598_2021_92511_MOESM1_ESM.pdf]

Optimal supply chains and power sector benefits of green hydrogen

Supplementary Information

Fabian Stöckl
German Institute for Economic Research (DIW Berlin), Germany, and
Technische Universität Berlin, Germany

Wolf-Peter Schill
German Institute for Economic Research (DIW Berlin), Germany, and
Energy Transition Hub, University of Melbourne, Australia
Corresponding author, `wschill@diw.de`

Alexander Zerrahn
German Institute for Economic Research (DIW Berlin), Germany

April 23, 2021

SI Supplementary Information

SI.1 Cost and emissions metrics

System Costs of Electricity (SCE) are the total power sector costs related to overall electricity generation. They include all investment, fixed, and variable power sector costs, but exclude the investment, fixed, and (non-electricity) variable costs of the hydrogen supply chains. Using the SCE, the benefits of integrating the power and hydrogen sectors are completely attributed to electricity generation. The SCE treat all electricity generation equally, irrespective of later consumption for conventional electricity demand, demand for hydrogen production and distribution, or losses in the transformation process.

Additional System Costs of Hydrogen (ASCH) are defined as the difference in total system costs between a scenario that includes hydrogen and the respective baseline without hydrogen demand, related to total hydrogen supply. The ASCH factor in the total power sector benefits of hydrogen supply. ASCH are not directly observable for market participants, but relevant from an energy sector planning perspective.

Average Provision Costs of Hydrogen (APCH), in contrast, sum the annualized costs of the hydrogen infrastructure and yearly electricity costs for hydrogen production, related to total hydrogen supply. Yearly electricity costs are the product of the hourly shadow prices of the model's energy balance and the hourly electricity demand along the hydrogen supply chain, summed up over all hours of a year. The APCH reflect a producer perspective (excluding taxes and fees that are potentially relevant in real-world settings). For alternative levelized costs of hydrogen (LCOH) concepts, see [11].

The **Additional System Emission Intensity of Hydrogen (ASEIH)** relates the overall difference of CO_2 emissions between a scenario with hydrogen and the respective baseline without hydrogen to the total hydrogen supply. Analogously to the ASCH, this metric takes the full power sector effects of hydrogen provision into account. Like ASCH, ASEIH are not directly observable in an actual market, but relevant from an energy sector planning perspective.

The alternative **Average Provision Emission Intensity of Hydrogen (APEIH)** metric is calculated by multiplying hourly average emission intensities of electricity generation with respective hourly electricity consumption for hydrogen supply at all steps of the supply chain (including compression, dehydrogenation etc.) and relating this to overall hydrogen provision. Analogously to the APCH, the APEIH assume a producer perspective.

SI.2 Sensitivities

We carry out a range of sensitivity calculations to explore how key parameter assumptions affect results. We investigate the effects of varying transportation distances, alternatively assuming that mass storage for small-scale on-site hydrogen supply is available, alternatively assuming that low-cost cavern storage for GH_2 is available as well as LH_2 storage without boil-off, and examine cost-free supply of heat as well as of transportation and storage infrastructure for LOHC.

SI.2.1 Transportation distance

Our baseline assumption for the transportation distance of hydrogen produced in large-scale facilities is 250 km, a value which we derive from previous analyses of the German case [21, 22]. Here, we examine the effects alternative transportation distance assumptions of 100 and 400 km. In general, a shorter/longer transportation distance increases/decreases the shares of large-scale hydrogen supply chains in the optimal solution, see Figures SI.1 and SI.2. Moreover, with a shorter transportation distance, large-scale technologies are now part of the optimal technology portfolio in some scenarios, while for a longer transportation distance, large-scale supply chains drop out in some scenarios.

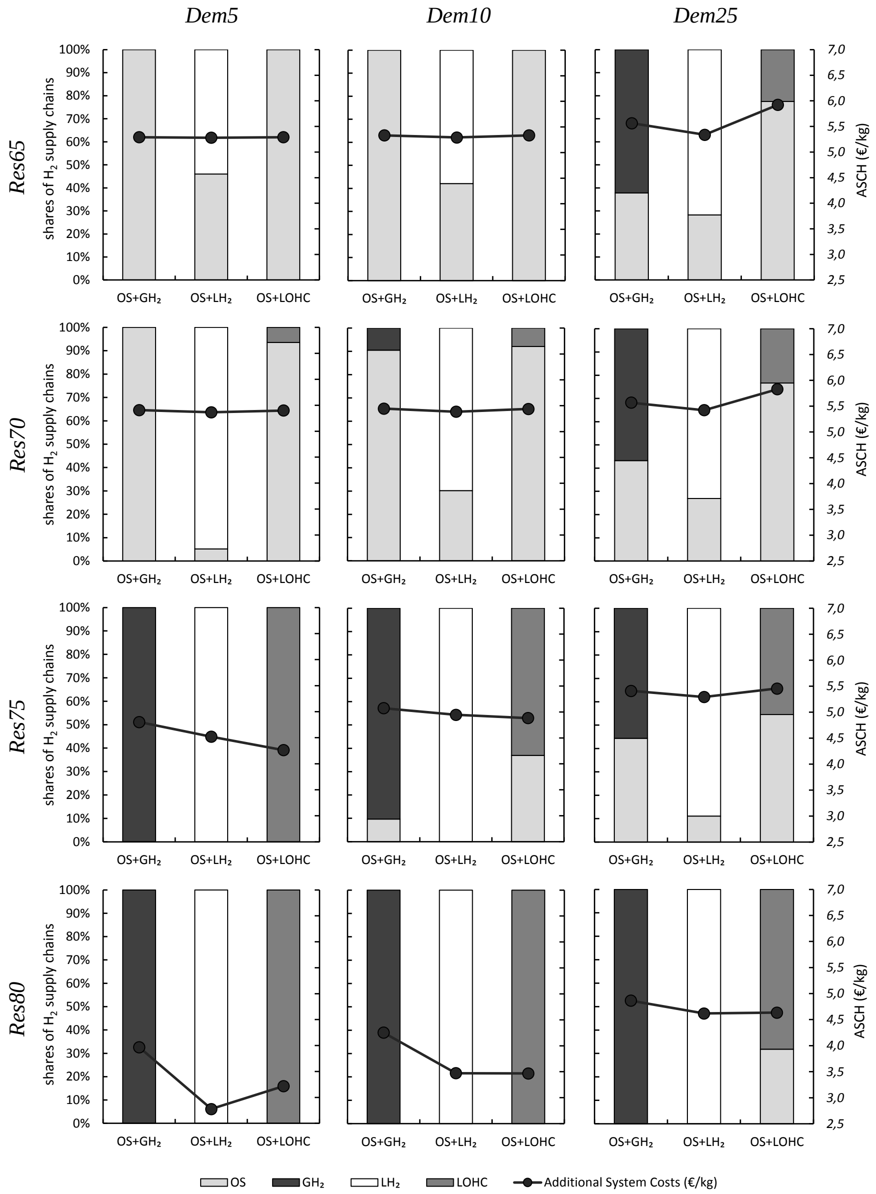

Figure SI.1: Optimal combinations of small-scale on-site and large-scale hydrogen supply chains and Additional System Costs of Hydrogen (ASCH) for different scenarios - sensitivity with 100 km transportation distance.

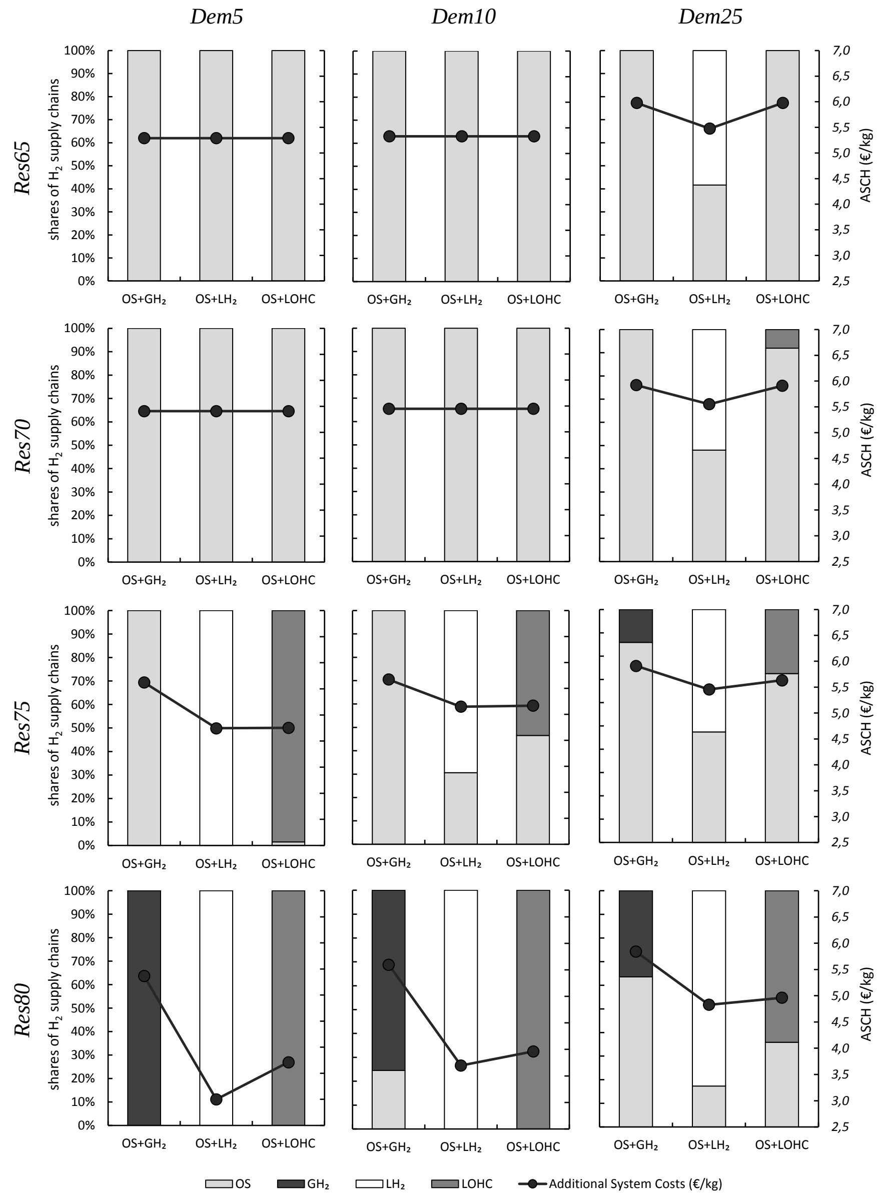

Figure SI.2: Optimal combinations of small-scale on-site and large-scale hydrogen supply chains and Additional System Costs of Hydrogen (ASCH) for different scenarios - sensitivity with 400 km transportation distance.

In general, a longer/shorter transportation distance increases/decreases the overall costs of the large-scale hydrogen supply chain. The spread in costs across supply chain combinations within scenarios tends to increase with transportation distance. Yet, the overall least-cost options are robust, with LH_2 as dominant large-scale supply chain in the optimal solution. Cost outcomes are fairly robust with respect to the transportation distance because the share of transportation-related costs in the overall costs of hydrogen provision are relatively small.

In more detail, a change in the average transportation distance has two effects on the costs of hydrogen supply. First, variable transportation costs (fuel and driver wage) are proportional to the transportation distance. For the sensitivity calculations with 400 km and 100 km transportation distances, the variable costs increase/decrease by 60 %. While the relative effect is the same for all three large-scale supply chains, the effect on absolute cost is highest for GH_2 and also more pronounced for LOHC than for LH_2, see Figure SI.3a.

Second, longer/shorter distances imply that each trailer is occupied for a longer/shorter time period. Consequently, the fleet capacity needs to be increased or can be reduced, respectively. Figure SI.3b shows transportation capacity investment costs per kg of hydrogen supplied through a specific supply chain averaged over all *Res-Dem*-scenarios. The pattern is identical to the one for variable costs, yet with less impact in absolute terms.

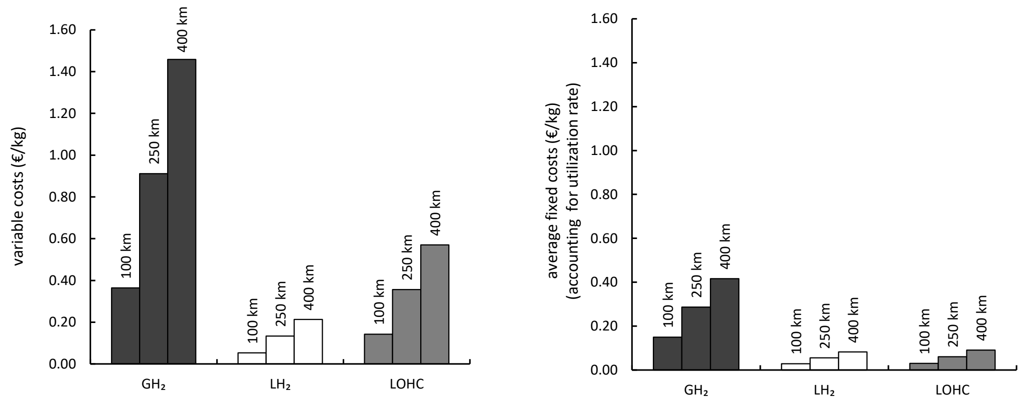

(a) (b)

Figure SI.3: Average transportation capacity investment costs and variable costs per kg of hydrogen supplied through the respective channel.

SI.2.2 Mass storage for small-scale on-site hydrogen supply

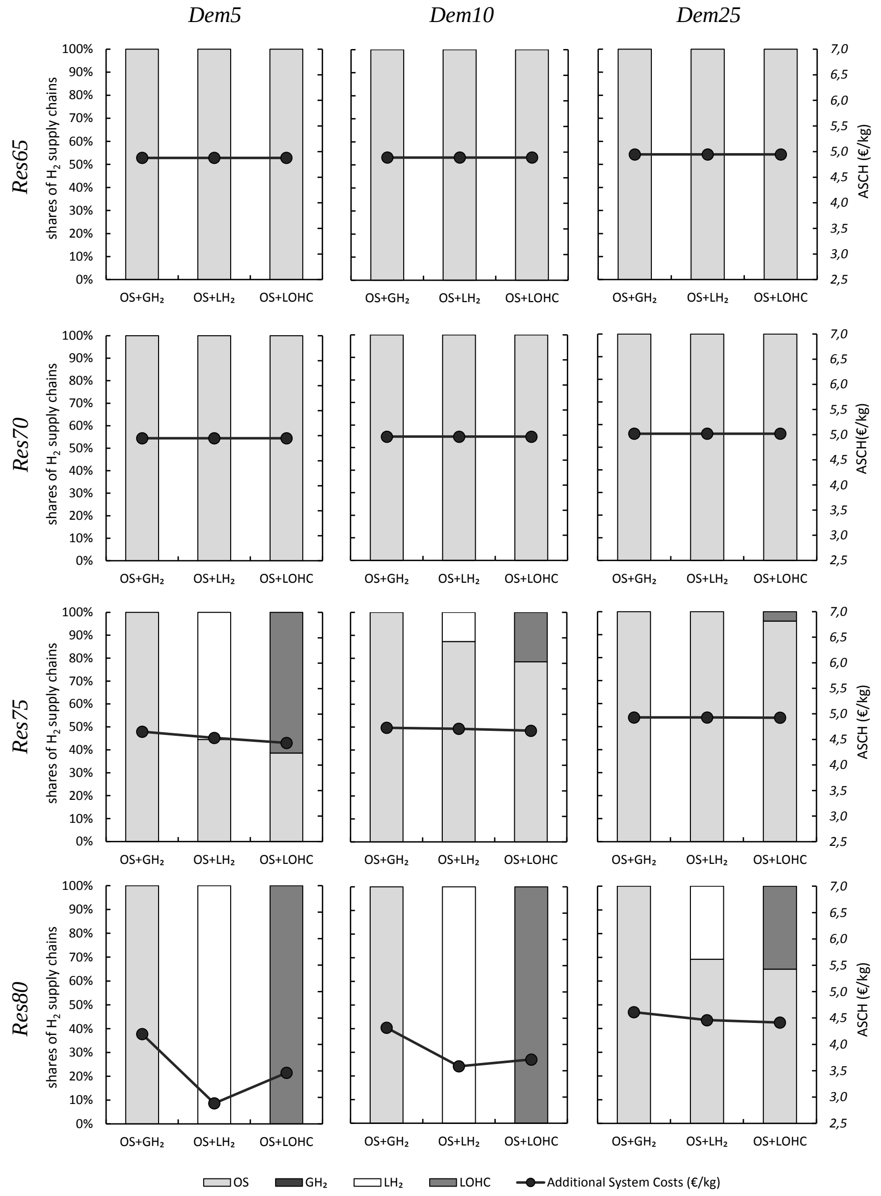

Figure SI.4: Optimal combinations of small-scale on-site and large-scale hydrogen supply chains and Additional System Costs of Hydrogen (ASCH) for different scenarios - sensitivity with mass storage available for small-scale on-site production.

Under baseline assumptions, mass hydrogen storage is not available at filling stations for small-scale supply because of space requirements and security concerns. Alternatively, we assume that relatively cheap mass storage at 250 bar can be deployed at filling stations, with the same techno-economic assumptions as for large-scale GH_2 storage. Table SI.13 gives an overview of the necessary changes with respect to compression processes and storage infrastructure.

Consequently, small-scale on-site production of hydrogen becomes more temporally flexible and loses its major disadvantage compared to large-scale production. Given that on-site hydrogen supply t filling stations is more energy-efficient, its share substantially increases for most supply-chain combinations and *Res*-*Dem*-scenarios (Figure SI.4), except for those with the highest renewable surpluses, i.e., *Res*80-*Dem*5 and *Res*80-*Dem*10, where all demand is still supplied by large-scale technologies. Here, large-scale production of LH_2 and LOHC still profits from a larger optimal storage size and the according flexibility. GH_2 produced in large-scale infrastructures drops out completely. As expected, with the additional flexibility option, the ASCH decrease slightly and the spread in costs between different supply chain combinations within each scenario rather decreases. Finally, the pattern of least-cost options across scenarios is robust, except for scenarios *Res*75-*Dem*25 and *Res*80-*Dem*25 where the cost-optimal technology portfolio now contains LOHC rather than LH_2.

SI.2.3 Cavern storage for GH_2

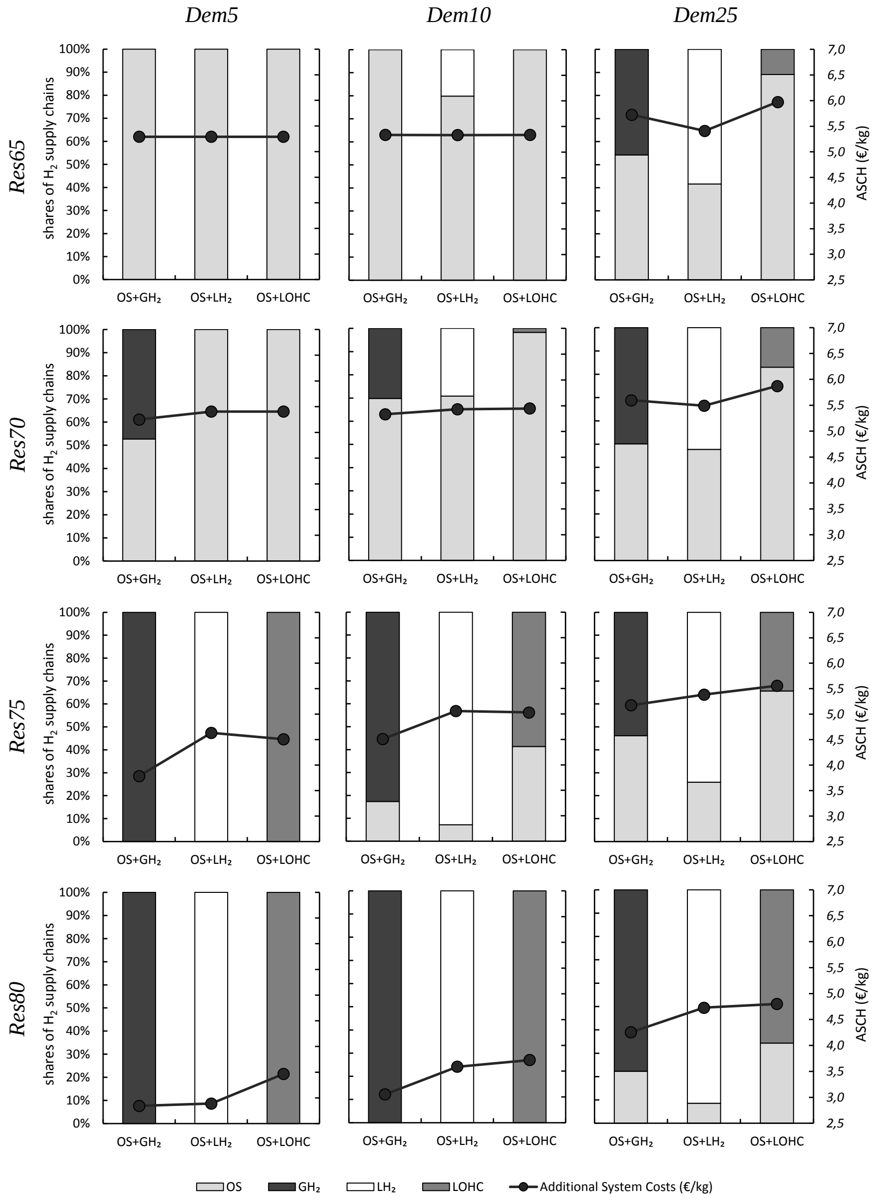

Figure SI.5: Optimal combinations of small-scale on-site and large-scale hydrogen supply chains and Additional System Costs of Hydrogen (ASCH) for different scenarios - sensitivity with cavern storage available for large-scale GH_2 production.

Low-cost cavern storage would provide flexibility for large-scale GH_2 production at very low costs of 3.5 €/kg, which is about one third of the costs of LOHC or LH_2 storage. Tables SI.4 and SI.6 list the altered requirements for compression processes.

If cavern storage is available, the share of large-scale GH_2 production increases substantially for all scenarios, see Figure SI.5. In contrast to the results under

default assumptions, the ASCH of the supply chain (DEC+)GH_2 are now lower than for the other options in most scenarios, especially if the share of renewable energy sources is high or H_2 demand is low. Moreover, Figure SI.6 illustrates that the use of cavern storage exhibits a seasonal pattern, as prevalent for LOHC in the baseline specification, yet with higher storage capacity due to low investment costs. Accordingly, the (non-)availability of cavern storage is a relevant driver of numerical model results.

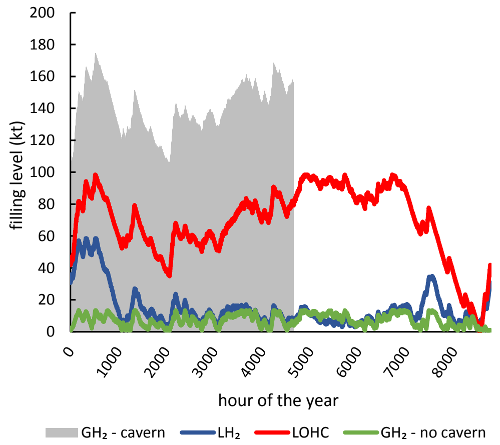

Figure SI.6: Temporal storage use patterns also including cavern storage for scenario *Res*80-*Dem*25

SI.2.4 No boil-off for LH_2

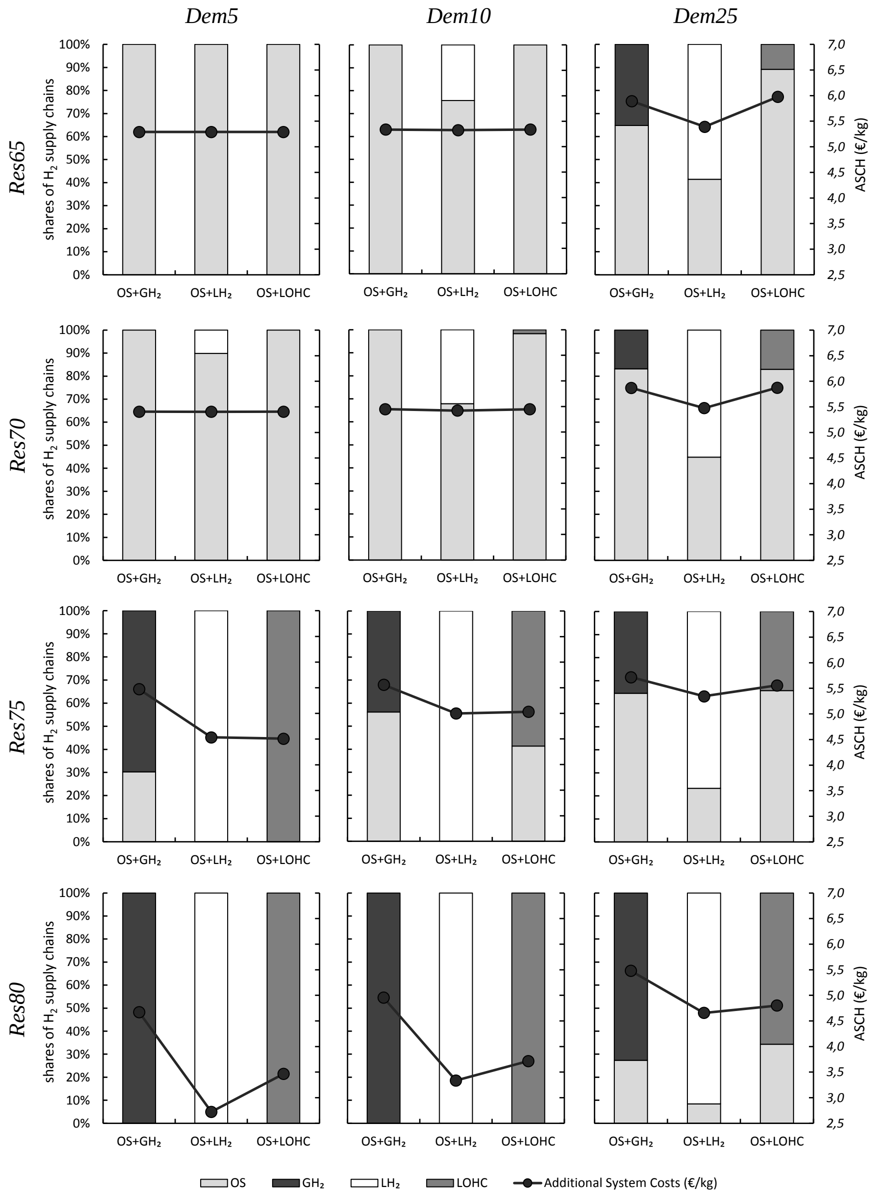

Figure SI.7: Optimal combinations of small-scale on-site and large-scale hydrogen supply chains and Additional System Costs of Hydrogen (ASCH) for different scenarios - sensitivity with no boil-off for LH_2 storage.

We assess the effects of LH_2 boil-off during storage and transportation by counter-factually setting it to zero. Figure SI.7 shows the results. The optimal shares of LH_2 compared to on-site hydrogen production at filling stations slightly increase in some cases, but effects are small. The average increase is 3.2 percentage points, and the largest increase is 10.2 percentage points in scenario *Res*70-*Dem*5. Likewise, the effect on H_2 costs is small, with an average cost reduction

of 1.8 % and a maximum decrease of 7.0 % in scenario *Res*80-*Dem*10. The pattern of least-cost options is robust with the combination containing LH_2 now additionally optimal for *Res*75-*Dem*10.

While the effect on costs and optimal technology shares is limited, LH_2 without boil-off is better suited as long-term or seasonal storage. Its use pattern changes substantially and resembles that of LOHC under default assumptions. Figure SI.8 exemplarily illustrates this point for scenario *Res*80-*Dem*25.

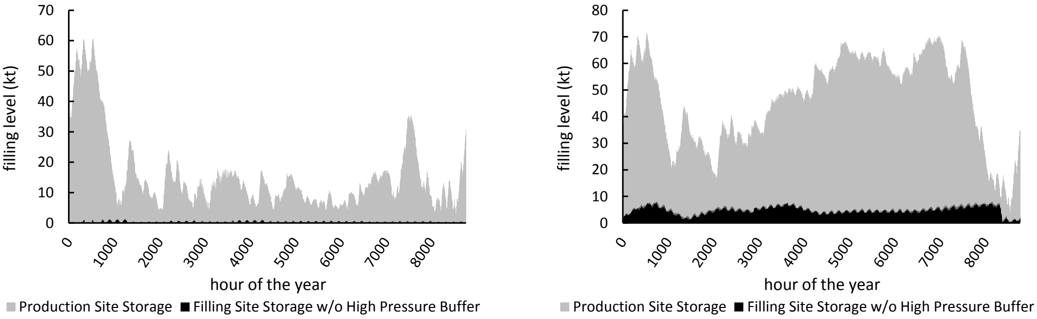

(a) With Boil-Off (b) Without Boil-Off

Figure SI.8: Temporal storage use patterns of LH_2 mass storage at the production site for scenario *Res*80-*Dem*25

Additionally, we find that LH_2 storage at the filling station becomes relatively more important if there is no boil-off. Under default assumptions, boil-off at the filling station was slightly higher than at the production site. Without boil-off, the two storage options are identical in terms of losses over time. Thus, the division of storage between the production and filling sites allows for a more efficient use of transportation capacities. This results in a decrease of transportation infrastructure costs of 5.5 % per kg of hydrogen in the scenario *Res*80-*Dem*25.

SI.2.5 Free heat supply for LOHC dehydrogenation

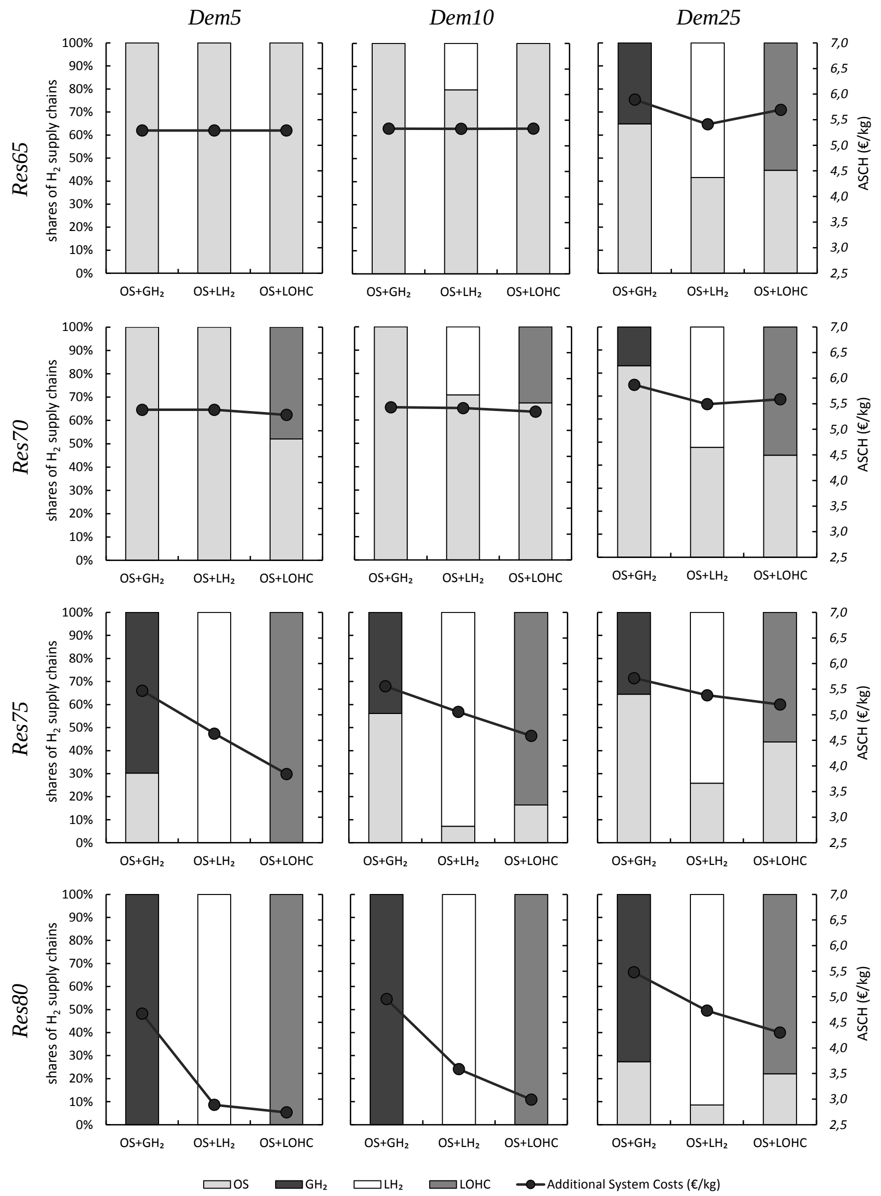

Figure SI.9: Optimal combinations of small-scale on-site and large-scale hydrogen supply chains and Additional System Costs of Hydrogen (ASCH) for different scenarios - sensitivity with free heat supply for dehydrogenation.

LOHC has a relatively high electricity demand for dehydrogenation, which is additionally temporally inflexible, that may hold back its extended use. We carry out a sensitivity calculation where the required heat is available free of costs, for instance, because industrial waste heat is available. Figure SI.9 shows the results. Compared to default assumptions, the share of LOHC increases in most scenarios. Also the ASCH for combinations of small-scale on-site electrolysis at

filling stations and LOHC decrease. With free heat supply, the LOHC supply chain is the least-cost solution for all scenarios with renewable shares of 75 % or 80 %.

SI.2.6 Free transportation and production-site storage infrastructure for LOHC

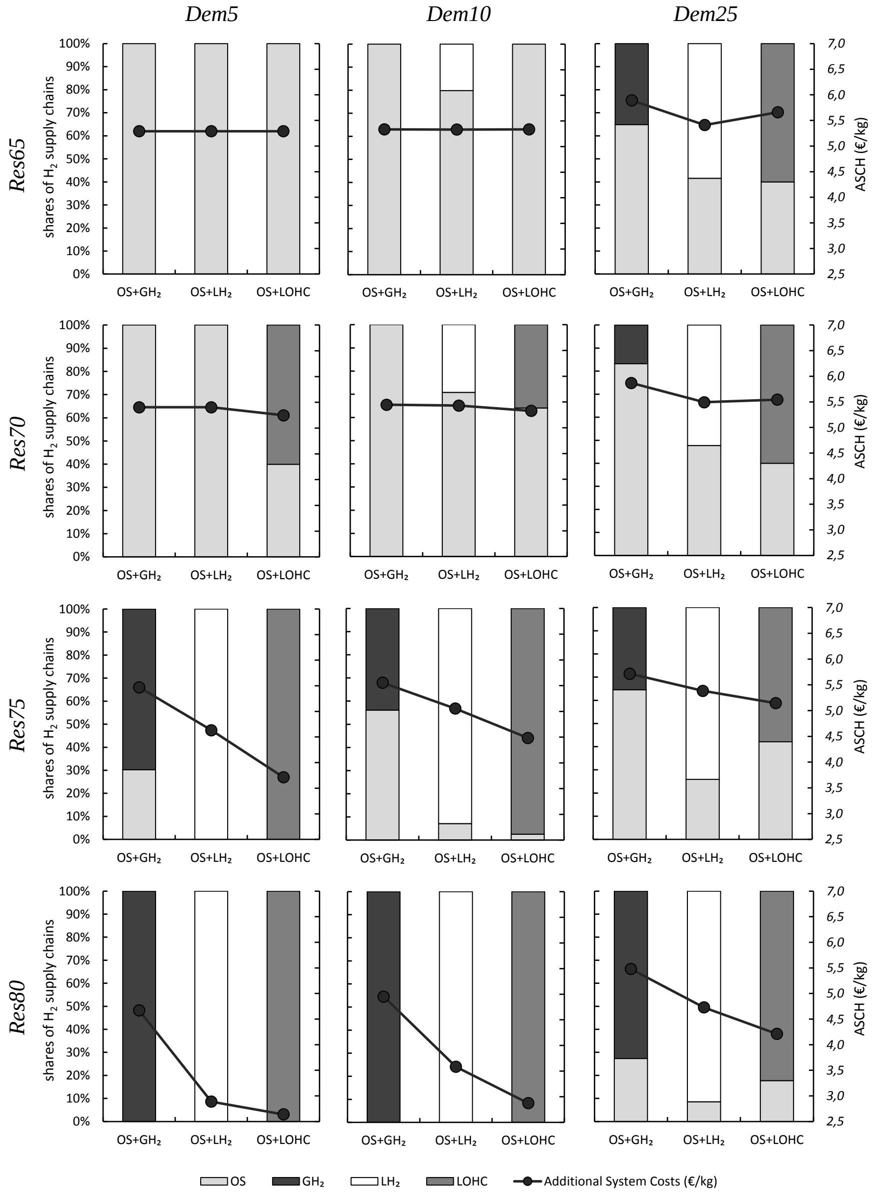

Figure SI.10: Optimal combinations of small-scale on-site and large-scale hydrogen supply chains and Additional System Costs of Hydrogen (ASCH) for different scenarios - sensitivity with free infrastructure for LOHC storage and transportation.

Proponents of LOHC argue that existing infrastructure may be used for the LOHC supply chain, especially storage at the production site and filling stations as well as transportation facilities [19]. To address this point in a sensitivity calculation, we assume that storage and transportation capacities do not incur

additional costs. Note that the expected lifetime of trailers is 12 years. The cost advantage of free transportation capacities would at most last for this time period. The results in Figure SI.10 show that the optimal share of LOHC increases only moderately in many scenarios. In contrast, the ASCH decrease substantially for all supply chains containing LOHC. As for the sensitivity calculation with free heat supply for dehydrogenation, the supply chain involving LOHC is the least-cost option in the scenarios with high renewable penetration also in this case (75 % or 80 %).

SI.3 Key power sector data

We apply our model to 2030 scenarios for Germany. To embed the analysis in a plausible mid-term future setting, electricity generation and storage capacities lean on the medium scenario B of the Grid Development Plan 2019 (*Netzentwicklungsplan*, NEP [3]), an official projection of the German electricity market that transmission system operators base their investments on.

NEP capacities for wind power, both onshore and offshore, solar PV, and battery storage serve as lower bounds for investments. NEP capacities for fossil plants, biomass plants, and run-of-river hydro power serve as upper bounds, where natural gas capacities are split evenly between combined- and open-cycle gas turbines. Coal capacities are largely in line with current German coal phase-out plans that target at most 9 and 8 GW lignite and hard coal by 2030, respectively. Investments for pumped storage are bounded from below by today's value and from above by the NEP value. Figure SI.11 summarizes the capacity bounds for the power sector.

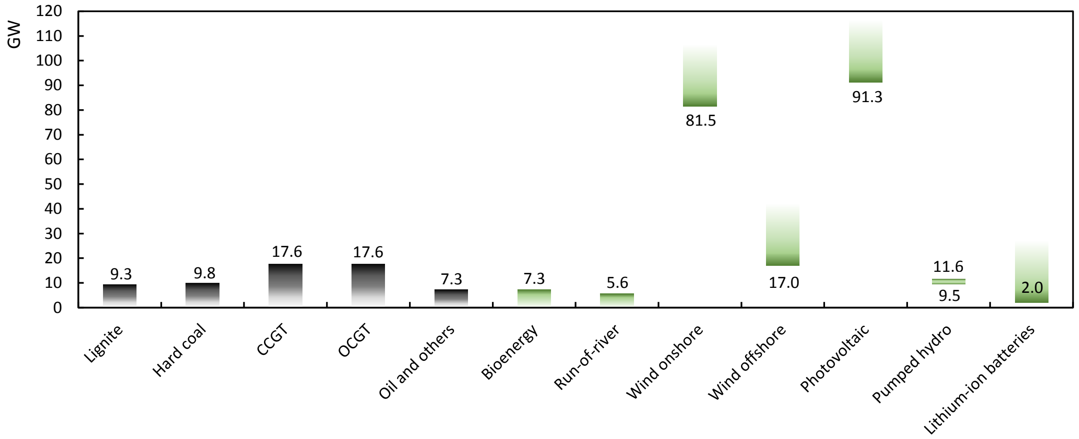

Figure SI.11: Lower and upper bounds for capacity investments in the power sector

Cost and technical parameters for power plants [25] and storage [16, 24] are based on established medium-term projections. Fuel costs and the CO_2 price of 29.4 €/t follow the middle NEP scenario B 2030. The hourly electricity load is representative for an average year and is taken from the Ten-Year Network Development Plan 2030 of the European Network of Transmission System Operators for Electricity [6]. Annual load sums up to around 550 Terawatt hours (TWh). Time series of hourly capacity factors for wind and PV are based on re-analysis data of the average weather year 2012 [18, 27].

All input data is available in a spreadsheet provided together with the open-source model [33].

SI.4 Key hydrogen sector data

In the following, we present key assumptions on the modeled hydrogen sector, including techno-economic parameters of hydrogen infrastructure as well as hydrogen demand. These are central drivers of the results. Full account of all input data is given in Section SI.4.3.

SI.4.1 Techno-economic parameters of H_2 infrastructure

PEM electrolysis is six percentage points more efficient than the ALK technology (71 % versus 66 %), but has about one-third higher specific investment costs (905 €/kW_{el} versus 688 €/kW_{el}). Moreover, based on industry data [12], we assume that investment costs of large-scale electrolysis are 20 % lower than those of small-scale on-site production at filling stations.

Cost differences also exist for hydrogen transportation. Trailers for GH_2 require high-pressure tubes (764 €/kg), for LH_2 an insulated tank (190 €/kg), and for LOHC only a simple standard tank (93 €/kg). Differences in variable costs are determined by the net loading capacity per trailer, where GH_2 is most expensive with 0.91 €/kg, compared to 0.36 €/kg and 0.13 €/kg for LOHC and LH_2, respectively. Fuel consumption (Diesel), wages for drivers, and (un-)loading times are assumed to be identical across all supply chains.

Investment costs for hydrogen storage are the central parameter that determines whether flexibility of a supply chain is economical. The costs of GH_2 storage at 250 bar (459 €/kg) is substantially higher than for LH_2 (14 €/kg) and LOHC (10 €/kg). LOHC has a degradation rate of 0.1 % per supply-cycle, entailing additional costs of 0.6 €/kg. We interpret these costs as LOHC rental rate. High-pressure gaseous (buffer) storage at the filling station is more expensive (612 €/kg) and requires a high minimum filling level in order to ensure pressure above 700 bar for dispensing. This reduces the effective available storage capacity further.

The techno-economic characteristics of the four hydrogen supply chains entail an efficiency-flexibility trade-off with respect to their electricity demand. Small-scale on-site production is relatively energy-efficient but needs to be almost on-time due to a lack of cheap storage options. The three large-scale supply chains are less efficient, but (partly) provide cheap storage options that allow to shift energy-intensive electrolysis to hours with high (renewable) electricity supply. Electricity demand for the remaining, inflexible processes to prepare stored hydrogen for dispensing at the filling station (recompression, cryo-compression, and evaporation or dehydrogenation), is comparably low. Figure SI.12 contrasts overall electricity demand with largely inflexible (i.e., non-shiftable) electricity demand at the filling station for different hydrogen supply chains across all scenarios. Within-channel deviations (min & max) are due to the choice of electrolysis technology and losses during storage.

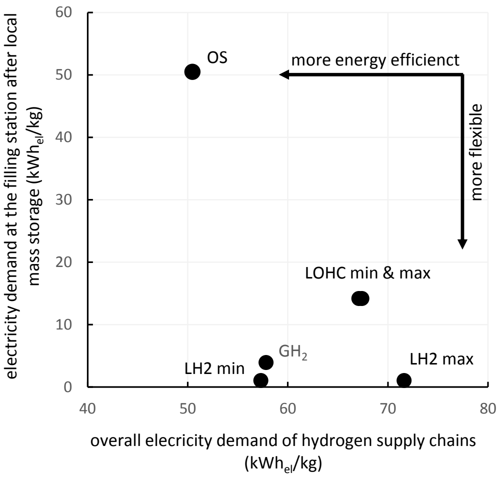

Figure SI.12: The (realized) efficiency-flexibility trade-off for different hydrogen supply chains across all scenarios.

SI.4.2 H_2 demand

H_2 demand for private and public road-based passenger transportation in Germany leans on a forecast for the year 2030 [26]. To convert gasoline and diesel consumption to H_2 demand [9], shares of fuel consumption for 2030 are assumed to be identical to those in 2017 [20]. Table SI.1 shows the resulting demands for the scenarios where 5 %, 10 %, or 25 % of private and public road-based passenger traffic in Germany in 2030 is fueled by hydrogen.

The hourly H_2 demand profile at the filling stations is assumed to be identical to today's for gasoline and diesel fuel. As data for Germany is not available, we resort to U.S. data for hourly and weekly [15] as well as for monthly [32] demand characteristics. Moreover, each filling station dispenses at most 1000 kg hydrogen per day [8]. This results in 976, 1952, and 4880 filling stations for the 5, 10, and 25 % demand scenarios, respectively.

Table SI.1: Traffic Data (2030 projection)

Scenario	H_2 demand	
	TWh	kt
5 %	9.053	271.610
10 %	18.160	543.220
25 %	45.265	1,358.050

Finally, depending on the average loading capacity and time a car spends at the filling station, a small amount needs to be added to the average costs of

hydrogen to cover dispenser costs (around 0.1 € for 5 kg per car with an average filling time of 7 min and a filling station capacity of 1000 kg/d, compare [22]). These costs are identical across all supply chain combinations and, thus, have no effect on their ranking.

SI.4.3 Data tables

In the following, we list all data and sources for techno-economic parameters concerning the H_2 infrastructure. As parameter projections for 2030 are scarce, except for electrolysis, we resort to values for currently existing or planned sites. All cost parameters are stated in euros (€). For conversion from U.S. dollar ($), we assume an exchange rate of one. As the literature on cost parameters does often not provide information on the reference year, we refrain from correcting for inflation. Unless stated otherwise, kg is always short for kg_{H_2}. To calculate electricity demand for compression and scale investment costs, we follow [21]. Pursuing a conservative approach, we always calculate energy demand for hydrogen compression for the least favorable initial pressure conditions. All data are in terms of the lower heating value (LHV). The costs of water for electrolysis are not taken into account in this analysis as they are negligible in Germany. Finally, OPEX are always stated as % of CAPEX.

Table SI.2: General assumptions

	Value
Average transportation distance (one-way) [21]	250 km
Average transportation speed [21]	50 km/h
Interest rate	4 %
Loading (LOHC) [7]	6.2 °weight-%
LOHC costs[a] [30]	4 €/kg_{LOHC}

a: LOHC has is a degradation rate of 2 × 0.1 % (hydrogenation & dehydrogenation) [30] per supply-cycle, entailing additional costs of 0.13 €/kg. We interpret these costs as LOHC rental rate.

Table SI.3: Assumptions for different electrolysis technologies for 2030

	ALK	PEM
CAPEX (€/kW_{el})[a] [12, 23]	550	724
OPEX (%) [1]	1.5	1.5
Depreciation period (a)[a, d] [1, 23]	10	10
Efficiency (%)[c] [1]	66	71
Pressure out (bar) [1, 4, 23]	30	30
Scale advantage (%)[b] [12]	20	20

a: Based on a 10 MW_{el} electrolysis system with 2 times the current R&D investment and production scale-up.
b: Cost advantage when scaling up from 2.2 MW_{el} to 10 MW_{el}. The output of a 2.2 MW_{el} and 10 MW_{el} electrolyzer with an efficiency of 68.5 % (the center of our assumptions for ALK and PEM) is equal to 45 kg/h and 206 kg/h, respectively.
c: At the system level, including power supply, system control, gas drying (purity at least 99.4 %). Excluding external compression, external purification, and hydrogen storage.
d: 60, 000 h operation at an utilization rate of 70 %.

Table SI.4: Assumptions for different storage preparation processes (production site)

	GH_2 (S)	GH_2 (L) [5]	$GH_2^{cav.}$ (L) [5]	LH_2 (L) [28]	LOHC (L) [7, 13, 14, 21, 30]
Activity	-	compression	compression	liquefaction	hydrogenation
CAPEX-base (€)	-	40,528	40,528	643,700	74,657 [7]
CAPEX-comparison	-	1 kW_{el}	1 kW_{el}	1 kg	1 kg
Scale	-	0.4603	0.4603	2/3	2/3
Ref.-Capacity (kg/h)	-	206	206	1030	1030
CAPEX-scaled (€/kg)[a]	-	2,923	2,672	63,739	7,392 [7]
OPEX (%)	-	4	4	4	4
Depreciation period (a)	-	15	15	30	20
Pressure in (bar)	-	30	30	30 (20 nec.)	30
Pressure out (bar)	-	250	180	2	-
Compression stages	-	2	2	-	-
Elec. Demand (kWh/kg)	-	1.707	1.402	6.78	0.37
Heat Demand (kWh/kg)	-	-	-	-	-8.9
Losses (%)	-	0.5	0.5	1.625	3

Abbreviations: cav.: cavern; (S): small-scale on-site supply chain; (L): large-scale supply chain
a: For 10 MW_{el} (206 kg/h) electrolysis capacity, the maximum daily throughput is almost 5 t of hydrogen. For non-stacked processes such as liquefaction and hydrogenation, we assume a throughput of 1030 kg/h which would be equal to the hydrogen production of a 50 MW_{el} electrolyzer.

Table SI.5: Assumptions for different storage types (production site)

	GH_2 (S)	GH_2 (L) [17]	$GH_2^{cav.}$ (L) [10]	LH_2 (L) [31]	LOHC (L) [21]
CAPEX-base (€)	-	450	3.5	13.31	10
CAPEX-comparison	-	1 kg	1 kg	1 kg	1 kg
Scale	-	1	1	1	1
CAPEX-scaled (€/kg)	-	450	3.5	13.31	10
OPEX (%) [21]	-	2	2.5 [29]	2	2
Depreciation period (a) [17]	-	20	30 [29]	20	20
Pressure range (bar)	-	15 - 250	60 - 180	-	-
Min. filling level (%)[a]	-	6	33.3	5	-
Boil-off (%/d) [2]	-	-	-	0.2	-
Storage bypass possibility	-	yes	yes	-	-

Abbreviations: cav.: cavern; (S): small-scale on-site supply chain; (L): large-scale supply chain
a: Calculated according to Boyle's law in order to maintain the minimum pressure required. For the cavern, minimum pressure is calculated dependent on the required amount of cushion gas.

Table SI.6: Assumptions for different transportation preparation processes

	GH_2 (S)	GH_2 (L) [5]	$GH_2^{cav.}$ (L) [5]	LH_2 (L)	LOHC (L)
Activity	-	compression	compression	overflow/pumping	
CAPEX-base (€)	-	6000	6000	-	-
CAPEX-comparison	-	1 kW_{el}	1 kW_{el}	-	-
Scale	-	1	1	-	-
Ref.-Capacity (kg/h)		720	720	-	-
CAPEX-scaled (€/kg)[a]	-	13,784	6,530	-	-
OPEX (%)	-	4	4	-	-
Depreciation period (a)	-	15	15	-	-
Min. Pressure in (bar)	-	15	60	-	-
Pressure out (bar)	-	250	250	-	-
Compression stages	-	2	2	-	-
Elec. demand (kWh/kg)	-	2.297	1.088	-	-
Losses (%)	-	0.5	0.5	-	-

Abbreviations: cav.: cavern; (S): small-scale on-site supply chain; (L): large-scale supply chain
a: 720 kg/h is equal to the trailer capacity. Thus, every compressor is required to have the capacity to load one trailer per hour.

Table SI.7: Assumptions for different transportation processes

	All [30]	GH_2 (L) [31]	LH_2 (L) [31]	LOHC (L) [21]
Function	tractor	trailer	trailer	trailer
CAPEX (€)[a, b]	223,031	518,400	865,260	150,000
Capacity (kg)	-	720	4,554	1,800
Net capacity (kg)[c]	-	676.8	4,326	1,620
CAPEX-net (€/kg)	-	763.93	190	92.59
OPEX (%)	12	2	2	2
Depreciation period (a) [30]	12	12	12	12
Losses (%/d) [2]	-	-	0.6	-
(Un-)/Loading time (h)	-	1 / 1	1 / 1	1 / 1

Abbreviations: (L): large-scale supply chain
a: CAPEX adjusted for a lifetime of 12 years with an interest rate of 4 %.
b: The average fuel consumption of a tractor is assumed to be 35 L/100 km [30]. Moreover, we assume a price of 1.30 €/L for diesel and an hourly wage of drivers of 35 €. Fuel is not covered by the CO_2 tax.
c: For GH_2, net-capacity is determined by the required outlet pressure. 5 % of LH_2 remain in the trailer to avoid heating up of the trailer-tank. For LOHC, a maximum discharge-depth of 90 % is assumed [7]. Thus, transportation capacity of actually usable hydrogen is below the total amount of bound hydrogen. For all other processes, issues linked to a discharge-depth below 100 % are ignored either because the effect on costs is negligible (storage, degradation) or because we assume a heat-recovery system being installed (dehydrogenation).

Table SI.8: Assumptions for different filling storage preparation processes (1st stage)

	GH_2 (S)	GH_2(L) [5]	LH_2 (L)	LOHC (L)
Activity	-	compression	overflow/pumping	
CAPEX-base (€)	-	40,035	-	-
CAPEX-comparison	-	1 kW_{el}	-	-
Scale	-	0.6038	-	-
Ref.-Capacity (kg/h)		676.8	-	-
CAPEX-scaled (€/kg)	-	4,744	-	-
OPEX (%)	-	4	-	-
Depreciation period (a)	-	15	-	-
Pressure in (bar)	-	15	-	-
Pressure out (bar)	-	250	-	-
Compression stages[21]	-	4	-	-
Elec. demand (kWh/kg)	-	2.105	-	-
Constraint (trailers/h)[a]	-	1	1	1
Losses (%)	-	0.5	2.5	-

Abbreviations: (S): small-scale on-site supply chain; (L): large-scale supply chain
a: Own assumption to avoid congestion at the filling station.

Table SI.9: Assumptions for different storage technologies (1st stage)

	GH_2 (S)	GH_2 (L) [17]	LH_2 (C) [31]	LOHC (L) [21]
CAPEX-base (€)	-	450	13.31	10
CAPEX-comparison	-	1 kg	1 kg	1 kg
Scale	-	1	1	1
CAPEX-scaled (€/kg)	-	450	13.31	10
OPEX (%) [21]	-	2	2	2
Depreciation period (a) [17]	-	20	20	20
Pressure range (bar)	-	15 - 250	-	-
Min. filling level (%)[a]	-	6	5	-
Boil-off (%/d) [2]	-	-	0.4	-
Storage bypass possibility	-	yes	-	-

Abbreviations: (S): small-scale on-site supply chain; (L): large-scale supply chain
a: Calculated according to Boyle's law in order to maintain the minimum pressure required.

Table SI.10: Assumptions for different filling storage preparation processes (2nd stage)

	GH_2 (S)	GH_2 (L)	LH_2 (L)	LH_2 (L)	LOHC (L)	LOHC (L)
	[5]	[5]	[5, 15]	[5, 15]	[7, 13, 14, 21, 30]	[5]
Activity	compression	compression	cryo-compr.-pump	evaporation	dehydrogenation	compression
CAPEX-base (€)	40,035	40,035	567.1 €/kg	900.9 €/kg	55,707	40,035
			+ 11,565 €	+ 2,389 €		
CAPEX-comparison	1 kW_{el}	1 kW_{el}	1 kg	1 kg	1 kg	1 kW_{el}
Scale	0.6038	0.6038	1	1	2/3	0.6038
Ref.-Capacity (kg/h)	45	45	45	45	45	45
CAPEX-scaled (€/kg)	17,014	19,070	824.1	954	15,662	22,220
OPEX (%)	4	4	4	1	4	4
Depreciation period (a)	10	10	10	10	20	10
Pressure in (bar)	30	15	2	-	-	5 [7]
Pressure out (bar)	950	950	-	950	5	950
Compression stages [21]	4	4	-	-	-	4
Elec. demand (kWh/kg)	2.947	3.559	0.1 [21]	0.6 [21]	-	4.585
Heat demand (kWh/kg)[a]	-	-	-	-	9.1	-
Losses (%)	0.5	0.5	-	-	1	0.5

Abbreviations: (S): small-scale on-site supply chain; (L): large-scale supply chain
a: 8.9 kWh/kg [14, 21] corrected for 97.5 % heat exchanger efficiency as described in [7].

Table SI.11: Assumptions for different storage technologies (2nd stage)

	All [31]
CAPEX-base (€)	600
CAPEX-comparison	1 kg
Scale	1
CAPEX-scaled (€/kg)	600
OPEX (%)	2 [21]
Depreciation period (a)	20 [17]
Pressure range (bar)	700 - 950
Min. filling level (%)[a]	74

a: Calculated according to Boyle's law in order to maintain the minimum pressure required.

Table SI.12: Assumptions for filling station equipment

	Refrigeration [5]	Dispenser [5]
CAPEX-base (€/pc.) [31]	70,000	60,000
OPEX (%)	2	1
Depreciation period (a)	15	10
Elec. demand (kWh/kg)	0.325	-
Max. temperature (°C)[a]	-40	-40

a: Hydrogen is dispensed to cars in gaseous form at 700 bar and pre-cooled to −40 °C in order to guarantee short filling times [5].

Table SI.13: Sensitivity: mass storage for small-scale on-site electrolysis

	GH_2 (S) [5]	GH_2 (S) [5]
Activity	compression (mass storage)	compression (high-pressure storage)
CAPEX-base (€)	40,035	40,035
CAPEX-comparison	1 kW_{el}	1 kW_{el}
Scale	0.6038	0.6038
Ref.-Capacity (kg/h)	45	45
CAPEX-scaled (€/kg)	11,972	17,014
OPEX (%)	4	4
Depreciation period (a)	15	10
Pressure in (bar)	30	30
Pressure out (bar)	250	950
Compression stages[21]	4	4
Elec. demand (kWh/kg)	1.654	2.947
Losses (%)	0.5	0.5

Abbreviations: (S): small-scale on-site supply chain

SI References

[1] L. Bertuccioli, A. Chan, D. Hart, F. Lehner, B. Madden, and E. Standen, “Development of Water Electrolysis in the European Union,” Commissioned by: Fuel Cells and Hydrogen Joint Undertaking, 2014, available at: https://www.fch.europa.eu/sites/default/files/study%20electrolyser_0-Logos_0_0.pdf [last accessed: Apr. 6, 2020].

[2] N. Bouwkamp, A. Burgunder, D. Casey, A. Elgowainy, L. Fisher, J. Merritt, E. Miller, A. Petitpas, G.and Rohatgi, N. Rustagi, J. Simnick, H. Soto, and J. Vickers, “Hydrogen Delivery Technical Team Roadmap,” Commissioned by: US DRIVE Partnership, 2017, available at: https://www.energy.gov/sites/prod/files/2017/08/f36/hdtt_roadmap_July2017.pdf [last accessed: Apr. 6, 2020].

[3] Bundesnetzagentur, “Genehmigung des Szenariorahmens 2019-2030,” 2018, available at: https://www.netzentwicklungsplan.de/sites/default/files/paragraphs-files/Szenariorahmen_2019-2030_Genehmigung_0_0.pdf [last accessed: Apr. 6, 2020].

[4] M. Carmo, D. L. Fritz, J. Mergel, and D. Stolten, “A Comprehensive Review on PEM Water Electrolysis,” *International Journal of Hydrogen Energy*, vol. 38, no. 12, pp. 4901–4934, 2013.

[5] A. Elgowainy, K. Reddi, M. Mintz, and D. Brown, “H2A Delivery Scenario Analysis, Model Version 3.0 (HDSAM 3.0),” 2015, available at: https://hdsam.es.anl.gov/index.php?content=hdsam [last accessed: Apr. 6, 2020].

[6] ENTSO-E, “Maps & Data for the Ten Year Network Development Plan 2018,” European Network of Transmission System Operators for Electricity, Tech. Rep., 2018, available at: https://tyndp.entsoe.eu/maps-data/ [last accessed: Apr. 6, 2020].

[7] M. Eypasch, M. Schimpe, A. Kanwar, T. Hartmann, S. Herzog, T. Frank, and T. Hamacher, “Model-Based Techno-Economic Evaluation of an Electricity Storage System Based on Liquid Organic Hydrogen Carriers,” *Applied Energy*, vol. 185, pp. 320–330, 2017.

[8] H2Mobility, “70MPa Hydrogen Refuelling Station Standardization - Function Description of Station Modules,” *Mimeo*, 2010.

[9] H. Hass, A. Huss, and H. Maas, “Tank-to-Wheels Report Version 4.a,” European Commission, Tech. Rep., 2014, available at: http://publications.jrc.ec.europa.eu/repository/bitstream/JRC85327/ttw_report_v4a_online.pdf [last accessed: Apr. 6, 2020].

[10] O. Kruck, F. Crotogino, R. Prelicz, and T. Rudolph, “Assessment of the Potential, the Actors and Relevant Business Cases for Large Scale and Seasonal Storage of Renewable Electricity by Hydrogen Underground Storage

in Europe," Commissioned by: Fuel Cells and Hydrogen Joint Undertaking, 2013, available at: http://hyunder.eu/wp-content/uploads/2016/01/D3.1_Overview-of-all-known-underground-storage-technologies.pdf [last accessed: Apr. 6, 2020].

[11] W. Kuckshinrichs and J. C. Koj, "Levelized Cost of Energy from Private and Social Perspectives: The Case of Improved Alkaline Water Electrolysis," *Journal of Cleaner Production*, vol. 203, pp. 619–632, 2018.

[12] H. G. Langås, "Large Scale Hydrogen Production," NEL, 2015, available at: https://www.sintef.no/contentassets/9b9c7b67d0dc4fbf9442143f1c52393c/9-hydrogen-production-in-large-scale-henning-g.-langas-nel-hydrogen.pdf [last accessed: Apr. 6, 2020].

[13] A. W. McClaine, K. Brown, and D. D. G. Bowen, "Magnesium Hydride Slurry: A Better Answer to Hydrogen Storage," *Journal of Energy Resources Technology*, vol. 137, no. 6, pp. 06 120 101–06 120 109, 2015.

[14] K. Müller, K. Stark, V. N. Emel'yanenko, M. A. Varfolomeev, D. H. Zaitsau, E. Shoifet, C. Schick, S. P. Verevkin, and W. Arlt, "Liquid Organic Hydrogen Carriers: Thermophysical and Thermochemical Studies of Benzyl- and Dibenzyl-toluene Derivatives," *Industrial & Engineering Chemistry Research*, vol. 54, no. 32, pp. 7967–7976, 2015.

[15] Nexant, Inc., Air Liquide, Argonne National Laboratory, Chevron Technology Venture, Gas Technology Institute, National Renewable Energy Laboratory, Pacific Northwest National Laboratory, and TIAX LLC, "H2A Hydrogen Delivery Infrastructure Analysis Models and Conventional Pathway Options Analysis Results - Interim Report," Commissioned by: US-DOE, 2008, available at: https://www.energy.gov/sites/prod/files/2014/03/f9/nexant_h2a.pdf [last accessed: Apr. 6, 2020].

[16] C. Pape, N. Gerhardt, P. A. Härtel, Scholz, T. Schwinn, R.and Drees, A. Maaz, J. Sprey, C. Breuer, A. Moser, F. Sailer, S. Reuter, and T. Müller, "Roadmap Speicher," Commissioned by: BMWi, 2014, available at: https://www.iee.fraunhofer.de/content/dam/iee/energiesystemtechnik/de/Dokumente/Studien-Reports/2014_Roadmap-Speicher-Langfassung.pdf [last accessed: Apr. 6, 2020].

[17] G. Parks, R. Boyd, J. Cornish, and R. Remick, "Hydrogen Station Compression, Storage, and Dispensing Technical Status and Costs: Systems Integration," NREL Technical Report, 2014, available at: https://www.hydrogen.energy.gov/pdfs/58564.pdf [last accessed: Apr. 6, 2020].

[18] S. Pfenninger and I. Staffell, "Long-Term Patterns of European PV Output Using 30 Years of Validated Hourly Reanalysis and Satellite Data," *Energy*, vol. 114, pp. 1251–1265, 2016.

[19] P. Preuster, C. Papp, and P. Wasserscheid, "Liquid Organic Hydrogen Carriers (LOHCs): Toward a Hydrogen-Free Hydrogen Economy," *Accounts of Chemical Research*, vol. 50, no. 1, pp. 74–85, 2017.

[20] S. Radke, *Verkehr in Zahlen 2017/2018*, BMVI, Ed. Hamburg: DVV Media Group, 2017.

[21] M. Reuß, T. Grube, M. Robinius, P. Preuster, P. Wasserscheid, and D. Stolten, "Seasonal Storage and Alternative Carriers: A Flexible Hydrogen Supply Chain Model," *Applied Energy*, vol. 200, pp. 290–302, 2017.

[22] P. Runge, C. Sölch, J. Albert, P. Wasserscheid, G. Zöttl, and V. Grimm, "Economic Comparison of Different Electric Fuels for Energy Scenarios in 2035," *Applied Energy*, vol. 233-234, pp. 1078–1093, 2019.

[23] O. Schmidt, A. Gambhir, I. Staffell, A. Hawkes, J. Nelson, and S. Few, "Future Cost and Performance of Water Electrolysis: An Expert Elicitation Study," *International Journal of Hydrogen Energy*, vol. 42, no. 52, pp. 30 470–30 492, 2017.

[24] O. Schmidt, A. Hawkes, A. Gambhir, and I. Staffell, "The Future Cost of Electrical Energy Storage Based on Experience Rates," *Nature Energy*, vol. 2, 2017.

[25] A. Schröder, F. Kunz, J. Meiss, R. Mendelevitch, and C. von Hirschhausen, "Current and Prospective Costs of Electricity Generation until 2050," DIW Berlin, Data Documentation 68, 2013, available at: https://www.diw.de/documents/publikationen/73/diw_01.c.424566.de/diw_datadoc_2013-068.pdf [last accessed: Apr. 6, 2020].

[26] M. Schubert, T. Kluth, G. Nebauer, R. Ratzenberger, S. Kotzagiorgis, B. Butz, W. Schneider, and M. Leible, "Verkehrsverflechtungsprognose 2030. Schlussbericht. Los 3: Erstellung der Prognose der deutschlandweiten Verkehrsverflechtungen unter Berücksichtigung des Luftverkehrs," Commissioned by: BMVI, 2014, available at: http://daten.clearingstelle-verkehr.de/276/1/verkehrsverflechtungsprognose-2030-schlussbericht-los-3.pdf [last accessed: Apr. 6, 2020].

[27] I. Staffell and S. Pfenninger, "Using Bias-Corrected Reanalysis to Simulate Current and Future Wind Power Output," *Energy*, vol. 114, pp. 1224–1239, 2016.

[28] K. Stolzenburg and R. Mubbala, "Integrated Design for Demonstration of Efficient Liquefaction of Hydrogen (IDEALHY)," Commissioned by: Fuel Cells and Hydrogen Joint Undertaking, 2013, available at: https://www.idealhy.eu/uploads/documents/IDEALHY_D3-16_Liquefaction_Report_web.pdf [last accessed: Apr. 6, 2020].

[29] K. Stolzenburg, R. Hamelmann, M. Wietschel, F. Genoese, J. Michaelis, J. Lehmann, A. Miege, S. Krause, C. Sponholz, S. Donadei, F. Crotogino, A. Acht, and P.-L. Horvath, "Integration von Wind-Wasserstoff-Systemen in das Energiesystem," Commissioned by: BMVI, 2014, available at: https://www.now-gmbh.de/content/1-aktuelles/1-presse/20140402-abschlussbericht-zur-integration-von-wind-wasserstoff-systemen-in-das-energiesystem-ist-veroeffentlicht/abschlussbericht_integration_von_wind-wasserstoff-systemen_in_das_energiesystem.pdf [last accessed: Apr. 6, 2020].

[30] D. Teichmann, W. Arlt, and P. Wasserscheid, "Liquid Organic Hydrogen Carriers as an Efficient Vector for the Transport and Storage of Renewable Energy," *International Journal of Hydrogen Energy*, vol. 37, no. 23, pp. 18 118–18 132, 2012.

[31] US-DOE, "Hydrogen Delivery," in *Fuel Cell Technologies Program Multi-Year Research, Development, and Demonstration Plan (MYRD&D Plan)*, US-DOE, Ed. US-DOE, 2015, ch. 3.2, available at: https://www.energy.gov/eere/fuelcells/downloads/fuel-cell-technologies-office-multi-year-research-development-and-22 [last accessed: Apr. 6, 2020 – subject to updates].

[32] US-EIA, "Prime Supplier Sales Volumes," 2018, available at: https://www.eia.gov/dnav/pet/pet_cons_prim_dcu_nus_m.htm [last accessed: Apr. 6, 2020].

[33] F. Stöckl, W.-P. Schill, and A. Zerrahn, "DIETER model version for the paper "Optimal supply chains and power sector benefits of green hydrogen"," Zenodo, 2020. https://doi.org/10.5281/zenodo.3693306
